# Early feeding leads to molecular maturation of the gut mucosal immune system in suckling piglets

**DOI:** 10.3389/fimmu.2023.1208891

**Published:** 2023-05-25

**Authors:** Raka Choudhury, Yuner Gu, J. Elizabeth Bolhuis, Michiel Kleerebezem

**Affiliations:** ^1^ Host-Microbe Interactomics Group, Department of Animal Sciences, Wageningen University & Research, Wageningen, Netherlands; ^2^ Adaptation Physiology Group, Department of Animal Sciences, Wageningen University & Research, Wageningen, Netherlands

**Keywords:** early life, dietary fiber, fibrous feed, microbiome, mucosal immune system, pig, transcriptomics

## Abstract

**Introduction:**

Diet-microbiota-host interactions are increasingly studied to comprehend their implications in host metabolism and overall health. Keeping in mind the importance of early life programming in shaping intestinal mucosal development, the pre-weaning period can be utilised to understand these interactions in suckling piglets. The objective of this study was to investigate the consequences of early life feeding on the time-resolved mucosal transcriptional program as well as mucosal morphology.

**Methods:**

A customised fibrous feed was provided to piglets (early-fed or EF group; 7 litters) from five days of age until weaning (29 days of age) in addition to sow’s milk, whereas control piglets (CON; 6 litters) suckled mother’s milk only. Rectal swabs, intestinal content, and mucosal tissues (jejunum, colon) were obtained pre- and post-weaning for microbiota analysis (16S amplicon sequencing) and host transcriptome analysis (RNA sequencing).

**Results:**

Early feeding accelerated both microbiota colonisation as well as host transcriptome, towards a more “mature state”, with a more pronounced response in colon compared to jejunum. Early feeding elicited the largest impact on the colon transcriptome just before weaning (compared to post-weaning time-points), exemplified by the modulation of genes involved in cholesterol and energy metabolism and immune response. The transcriptional impact of early feeding persisted during the first days post-weaning and was highlighted by a stronger mucosal response to the weaning stress, via pronounced activation of barrier repair reactions, which is a combination of immune activation, epithelial migration and “wound-repair” like processes, compared to the CON piglets.

**Discussion:**

Our study demonstrates the potential of early life nutrition in neonatal piglets as a means to support their intestinal development during the suckling period, and to improve adaptation during the weaning transition.

## Introduction

In commercial pig farming, after weaning piglets have to deal with multiple stressors including separation from the sow, a sudden dietary shift from milk to solid feed as well as a novel environment and often unfamiliar pen-mates, leading to a transient low feed intake or anorexia, intestinal inflammation, and an unbalanced gut microbiota or dysbiosis (1–4). Weaning stress in piglets is thus characterised by a multitude of changes in the intestinal physiology and function and is often accompanied by post-weaning diarrhoea. Several studies have reported decreased intestinal digestive enzyme activities, damaged tight junction proteins, impaired immune response and barrier function, as well as altered mucosal morphometry, especially during the first week after weaning (5–10). For instance, alterations in intestinal mucosal architecture typically occurs post-weaning, often characterised by lowered villus length and deepened crypt depth (3, 11). Furthermore, the process of weaning triggers transcriptional changes in the intestinal mucosa of pigs, mostly related to oxidative stress and immune activation (12–14). It is therefore important to employ appropriate nutrition and management strategies to minimise the adverse effects of weaning stress, aiming to improve animal health and welfare during the weaning transition, which may also have a positive health-impact later in life. Furthermore, pigs can be utilized as a translational model (15–18) to study the developmental consequences of early life perturbation, and/or nutritional studies in humans. Such proposition is based on the striking similarity of pigs and humans with respect to their anatomical- (cardiovascular and urinary system, skin, brain) and functional-characteristics (gastrointestinal and immune system), compared to the more commonly used rodent models (16, 19–24). For instance, it was reported that the porcine immune system more closely resembles humans for >80% of analyzed parameters (*vs.* <10% in mice) (25).

At the time of weaning in commercial pig-husbandry systems (around 3-4 weeks of age), the gastrointestinal tract of the piglets is still developing (26), including drastic adaptations in microbiota colonisation and the mucosal immune system (10, 27). The pre-weaning period in early life, thus provides a “window of opportunity” to modulate and support the host gastrointestinal function of the young piglet (28, 29). Inclusion of fibres in pre-weaning diet (instead of milk-based creep feed) may positively influence gut function and animal health, and thus can be used as a contemporary pig management strategy to change the structure of the gut microbial communities (30). Dietary fibres are known to influence gut microbiota *via* microbial fermentation of fibres leading to production of short chain fatty acid (SCFAs) such as butyrate, acetate and propionate (31). Moreover, SCFAs are recognised to influence intestinal functions such as the enhancement of the intestinal barrier function, as well as mucosal immune system development (14, 32, 33).

Studies reporting on the host mucosal transcriptome response to dietary fibres are scarce (34, 35), especially in piglets during the pre-weaning period. The studies that assessed the dietary fibre-microbiota-host interaction mostly employed growing pigs or were performed in alternative animal models, like mice (36–41). Previously we have reported that early feeding of fibrous feed leads to acceleration of the intestinal microbiota composition towards a more mature microbiome in suckling piglets (42). In the present study, we expand this work to evaluate the influence of early feeding and the coinciding microbiota changes on the development of the host mucosa. We therefore provided fibrous feed pre-weaning (from five days of age) and studied its impact on the host mucosal transcriptome patterns, mucosal morphology and proliferation at the moment of weaning (29 days of age). In addition, similar analyses at two time-points post weaning (3 and 21 days after weaning) enabled us to investigate the post-weaning development and evaluate the persistence of the effects of early life feeding.

## Materials and methods

### Animals, experimental design and sampling

The Animal Care and Use committee of Wageningen University & Research (Wageningen, The Netherlands) approved the protocol of the experiment (AVD104002016515). The protocol is in accordance with the Dutch law on animal experimentation, which complies with the European Directive 2010/63/EU on the protection of animals used for scientific purposes.

**Figure 1:**
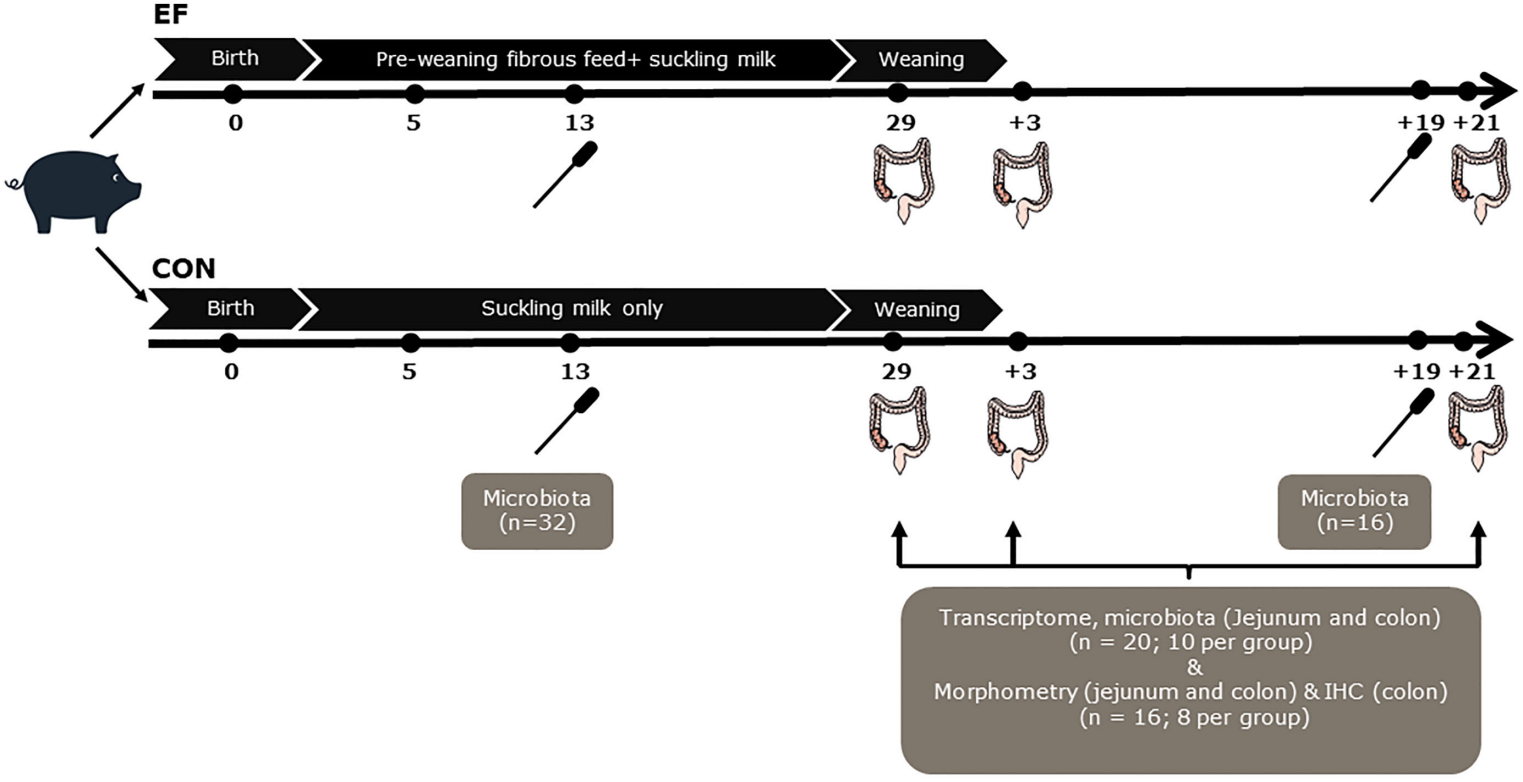
Schematic experimental design and sampling. Rectal swabs were collected at day 13 (n=32; 16 per group) and d+19 (n=16; 8 per group) for pre-and post-weaning microbiota analysis. Although the number of samples are not equal, the distinctive pre- and post-weaning microbiome composition are in line with our previous study (42), and were confirmed in principal component analysis (data not shown)]. Jejunal and colon samples were collected from the sacrificed piglets (n=20; 10 per group) to perform transcriptome and microbiota analyses at the time of weaning (day29), 3 days post-weaning (day+3) and 21 days post-weaning (day+21). Notably, luminal samples at day+21 were not included for microbiota analysis, instead the rectal swab samples at day+19 were used as a proxy, to reflect the ‘relatively mature’ post-weaning microbiota composition. Morphometric measurements (jejunal and colon samples; n=8 per group) and immunohistochemical (IHC) staining (colon samples; n=8 per group) were also performed at day29, day+3 and day+21.

The experiment was conducted with thirteen multiparous Topigs-20 sows (Parity 2-8) and their new-born piglets (Tempo x Topigs-20), housed at research facility Carus (Wageningen University & Research, The Netherlands) (**Figure 1**). The litters (n =13; 155 piglets in total) were divided into two treatment groups, early-fed or EF group (n=7 litters; 84 piglets; 41 female and 43 male) and control or CON group (n=6 litters; 71 piglets; 35 female and 36 male), balanced for sow parity, body weight and genetic background. Within two days after birth, the litter size was set to a maximum of 15 piglets per litter with no cross-fostering. The new-born piglets were cohoused with their mother and littermates till weaning (29 days of age) and received ear tags for individual identification and an iron injection, standard to pig husbandry practice. Piglets belonging to the EF group were given access to customised fibrous feed *ad libitum* (**Supplementary Table 1**) from 5 days of age in addition to suckling sow’s milk, whereas the CON group suckled sow’s milk only. To stimulate foraging/feeding behaviour in young piglets, two strategies were incorporated: (a) offering the feed in a play-feeder (enriched with non-consumable playing materials) and (b) learning from the mother (sow) during the first two weeks of life, foraging together for 10 mins (twice) every day, *via* floor/scatter feeding (close to the feeder location). Briefly, the feed included sugarbeet pulp (4%), oat hulls (4%), inulin (4%), galacto-oligosaccharides (5%) and resistant starch (4%) as fibrous ingredients. A subset of piglets (n=72; 36 per treatment group) were weaned at 29 days of age and followed until 21 days post-weaning. At weaning, piglets were mixed within the same treatment group and housed in separate pens, having three piglets per pen. After weaning, all piglets had *ad libitum* access to a standard weaner diet (**Supplementary Table 2**), mixed and pelleted by Research Diet Services (Wijk bij Duurstede, The Netherlands).

Rectal swabs were collected at pre- (13 days of age [day13]; n = 32) and post-weaning (19 days after weaning [day+19]; n = 16) time-points for microbiota analysis. This was done by inserting a sterile cotton swab (Puritan Medical, Guilford, ME USA; Cat Number-25-3306-U) 20–30 mm into the rectum and rotating the swab against the bowel wall for a minute before placing it into a 5ml eppendorf tube. The samples were put on ice immediately after collection, transported to the laboratory and stored at −20°C until further processing. Intestinal content and mucosal tissue samples were collected at three time-points (just before weaning [day29], 3 days after weaning [day+3] and three weeks after weaning [day+21]) following the sacrifice of a subset of piglets (10 piglets per treatment per time-point). The selection of sacrificed piglets at pre- and post-weaning time-points was made by the following criteria: a) no antibiotic treatment b) close to mean body weight of the litter c) moderate to good eaters (scan sampling for EF piglets; see below) d) no pre-weaning diarrhoea e) gender balanced for treatment. Instantaneous 1-min scan sampling (6 hours per day) on three time-points pre-weaning (15, 22 and 26 days of age), was used to determine “eaters”, allowing the selection of EF piglets that were sacrificed for bio-sampling. All piglets were scanned once each minute and the observer recorded whether piglets were eating or chewing feed from the trough or floor. Just before weaning, the data from all three observation days (60*6*3 = 1080 scans) were combined and subsequently piglets were classified into “good”, “moderate” and “bad” eaters (i.e., found to be eating or chewing feed) at least once, in 3, 2, 1 day (s) out of 3 days respectively (data not shown).

Piglets were euthanised by intravenous injection of 20% sodium pentobarbital (EUTHASOL^®^, 500 mg/ml, AST Farma B.V., Oudewater, The Netherlands). After euthanasia, the gastrointestinal tract was removed and dissected immediately to collect intestinal segments (~25cm) from jejunum (1.5 metres from duodenal-jejunal flexure) and colon (mid-spiral colon). For host mucosal tissue transcriptome analyses, tissue samples (2-3 cm) were collected from the intestinal segments in RNAlater™ and snap frozen in liquid nitrogen. For microbiota analysis, jejunal and colon luminal contents were collected from the adjacent part of the segment (approximately 10cm) in a 5ml eppendorf tube and immediately frozen in liquid nitrogen. Lastly, the adjacent posterior tissue part (whole tissue; 2 cm) was collected in 4% paraformaldehyde (PFA) for histology and immunohistochemistry. All intestinal samples were stored at -80°C until further processing.

### DNA extraction and 16S rRNA gene based amplicon sequencing

DNA was extracted from rectal swabs and intestinal content by the repeated bead beating method (43) using QIAamp PowerFecal^®^ DNA Kit (Qiagen, Hilden, Germany) according to manufacturer’s instructions. PowerBead solution (750 μl) was added to the 5ml eppendorf tube (containing rectal swab) and mixed well to obtain the extracted swab solution that was used as a starting material for DNA extraction. For jejunum and colon samples, approximately 200 mg of luminal content (wet weight) was used for microbial DNA extraction. The quality and quantity of extracted DNA samples were checked by gel electrophoresis (only representative samples) and Qubit™ 4.0 Fluorometer (Thermo Fisher Scientific, Wilmington, DE USA), respectively.

Library construction of the V3-V4 hypervariable region (from 16S rRNA gene) followed by sequencing on an Illumina HiSeq 2500 platform (paired end reads; 2*250 bp) were performed at Novogene (Novogene Co. LTD, China). Amplicons of the V3-V4 hypervariable region of the 16S rRNA gene, were generated using the primer set 341F/806R (341F: 5′-CCTAYGGGRBGCASCAG-3′, 806R: 5′-GGACTACNNGGGTATCTAAT-3′). All PCR reactions were carried out with Phusion^®^ High-Fidelity PCR Master Mix (New England Biolabs Ltd., Ipswich, USA), according to standard protocols at Novogene. The PCR products were mixed with the same volume of 1x loading buffer (contained SYBR green) and were detected by electrophoresis on 2% agarose gel. Samples with a bright band between 400–450 bp were used for library construction. Prior to library preparation, the PCR products were mixed in equimolar ratio and purified using Qiagen Gel Extraction Kit (Qiagen, Hilden, Germany). Sequencing libraries were constructed using TruSeq DNA PCR-Free Sample Preparation Kit (Illumina Inc., San Diego, USA), according to the manufacturer’s instructions. Subsequently, after in-house quality check at Novogene, the sample-specific barcodes and primer sequences were trimmed from the Illumina raw reads.

### 16S sequencing data analysis

The trimmed paired end reads were imported into the CLC Genomics Workbench version 11.01 and were processed using the CLC Microbial Genomics Module version 2.5.1 (CLC bio, Arhus, Denmark). The paired end reads were merged into one high quality representative sequence using CLC default parameters (Mismatch cost = 1, Minimum score = 40, Gap Cost = 4, Maximum unaligned end mismatches = 5). The sequences were then clustered into operational taxonomic unit (OTUs) at 97% identity threshold, followed by taxonomic annotation using SILVA database v132 (released on Dec 13, 2017) (44). Multivariate redundancy analysis (RDA) was employed to identify microbial signatures in different time-points or treatment groups using CANOCO 5 (Microcomputer Power, Ithaca, NY, USA) according to manufacturer’s instructions (45). Additionally, the linear discriminant analysis effect size (LEfSe) algorithm (46) was used to characterize the microbial differences between groups. Principal coordinate analysis (squared Bray Curtis distance) was employed to assess microbiota maturation in colon (day29 and day+3) by comparing to the relatively ‘mature’ post-weaning time-point (day+19) in rectal swabs.

### RNA-seq library preparation and sequencing

Total RNA was extracted from the jejunal and colonic tissue samples using RNeasy Mini Kit (Qiagen, Hilden, Germany), according to the manufacturer’s recommendations. RNA quantity and quality were assessed using Qubit™ 4.0 Fluorometer (Thermo Fisher Scientific, Wilmington, DE USA), and the Agilent 2200 TapeStation system (Santa Clara, CA, USA). All RNA samples were found to be suitable for RNA sequencing, based on the minimum requirements that the sample showed intact 18S and 28S ribosomal RNA bands, and had a RIN (RNA Integrity Number) above 8.0.

The RNA samples were transferred to Novogene (Novogene Co. LTD, China) for library preparation and RNA sequencing. Briefly, 1 µg total RNA in 50 µl was used for library preparation using NEBNext^®^ Ultra Directional RNA Library Prep Kit for Illumina^®^ (NEB, Ipswich, MA, US) following the manufacturer’s protocol. After RNA quality verification, mRNA was enriched using NEBNext Oligo d(T), fragmented randomly and first strand cDNA was synthesised using random primers. Second Strand cDNA was synthesised using dNTPs (with dUTP replacing dTTP). The double-stranded cDNA was then purified using Agencourt AMPure XP Beads (Beckman Coulter, Beverly, MA, USA), followed by end repair of cDNA library and adaptor ligation. The adaptor ligated DNA was PCR enriched and the PCR products were purified by Agencourt AMPure XP Beads. The library quality was assessed by Agilent 2100 Bioanalyzer (Agilent, Santa Clara, CA, USA), Qubit 2.0 fluorometer (Thermo Fisher Scientific, Waltham, MA USA) and qPCR (iCycler, BioRadLaboratories, Hercules, CA, USA). The libraries were sequenced on Illumina Novaseq6000 (Illumina, San Diego, CA, USA) by Novogene (Novogene Co. LTD, China) at 6 Gb raw data/sample with 150 bp paired-end reads. The raw reads (in fastq format) were processed to remove adapter sequences, using in house scripts.

### Differential expression analysis and biological interpretation of transcriptome data

Sequencing reads were imported in the CLC Genomic Workbench (QIAGEN, Aarhus, Denmark) according to their workflow, mapped to the *Sus scrofa* 11.1 reference genome, resulting in read counts per gene which was used for downstream analysis. Differential gene expression (DGE) analyses of the treatment groups were performed using ‘Empirical analysis of DGE’ tool in CLC workbench, that employs the ‘Exact test’ (47) incorporated in the EdgeR bioconductor package (48). Lowly expressed genes (< 5 counts per million [CPM], in more than 80% of the samples) were excluded from further analysis according to EdgeR pipeline (49). The web-based galaxy platform (https://usegalaxy.org/; RNAseq tool in the EdgeR package) was used to obtain the normalised counts (logCPM) of the filtered gene list (~12000 genes). Further, the unknown Ensemble gene IDs (*Sus scrofa*) in the list were converted into gene names using g:Profiler (g:Convert tool) (50), with 435 (colon) and 416 (jejunum) out of 1332 of these unknown IDs attaining gene annotation (**Supplementary Table 3**). Using this (filtered, normalised) gene table, principal component analysis (PCA), principal co-ordinate analysis (PCoA, squared Bray Curtis distance) and principal response curve (PRC) analyses were performed in CANOCO 5 (Microcomputer Power, Ithaca, NY, USA) (45). Principal response curve analyses detected temporal changes in transcriptome and its interaction with early feeding treatment. In addition, the significance of the interaction was tested using MonteCarlo permutation test (499 permutations). Among the total of 120 samples, two were classified as outliers (51, 52), employing modified Z scores of summed PC components (**Supplementary Figure 1A**) and removed from further analysis.

To identify canonical pathways associated with up- and down-regulated genes in the EF group relative to the CON group, Ingenuity Pathway Analysis (IPA; Ingenuity Systems, Redwood city, CA, USA) was employed, which uses curated information from the Ingenuity Knowledge Base (www.ingenuity.com). IPA provides statistical assessment (based on Fisher’s exact test) of biological pathway enrichment, by determining whether there are non-random associations. The statistical test calculates the probability of genes associated with a pathway (from the dataset) relative to the total number of genes that define the canonical pathway within the IPA knowledge base. The list of differentially expressed genes was uploaded into the IPA software, containing gene identifiers and corresponding fold change (FC; absolute FC > 1.2) and *P* values (< 0. 05). Canonical pathways identified in IPA, having logP value ≥ 1.3 (or *P* < 0.05; enrichment score from Fisher’s exact test) and an absolute Z score ≥ 2 (assessing the match of observed and predicted up/down regulation patterns) (53), were subsequently visualised in GraphPad Software 8.1.1 (California, USA). Following primary DGE analysis (i.e., the convergence of post-weaning transcriptome at day+21; see results), the gene expression changes over time were evaluated by normalising expression values (logCPM; per group per time-point) by scaling to the mean expression value of day+21 (irrespective of treatment), and visualised in Multiple Experiment Viewer (MeV) version 4.9.0 (http://mev.tm4.org/#/welcome) (54). In addition, unsupervised hierarchical clustering (euclidean distance) of the identified genes was performed in MeV, for group comparison over time.

To assess the functional interactions among pathway-associated genes, the Reactome FIviz (functional interaction) application (55) in Cytoscape 3.7.1 (56) was employed. The genes corresponding to enriched pathways in EF group (identified in IPA; unique to one or more time-point(s)) were used to build the functional networks (‘Gene Set Analysis’ tool) utilising information from the Reactome FI database (57), which is an expert-curated, peer reviewed database of human biological pathways. Prior to building the network, the porcine gene IDs needed to be converted into their human homologues using g:Profiler (g:Orth tool) (50).

### Histology analysis and immunohistochemical staining of colonic proliferating cells

Intestinal tissues (jejunum and colon) collected at three time-points day29, day+3 and day+21, were fixed in 4% paraformaldehyde (PFA), and then dehydrated and embedded in paraffin blocks (8 animals per treatment per time-point per location). Histological and immunohistochemical staining were performed as previously described (58). Briefly, 5 µm sections were cut with a Accu-Cut^®^ SRM™ 200 Rotary Microtome (Sakura Finetek Europe B.V., Alphen aan de Rijn, The Netherlands), deparaffinized, hydrated, stained with Haematoxylin-eosin (H&E) and examined using a Leica DM6 B microscope (Leica Microsystems Ltd. CH9435 Heerbrugg). Images (5x magnification) were processed with LAS X software (Leica Microsystems Inc., Buffalo Grove, IL, USA) to measure villus length and crypt depth (μm) from tissue sections (60 measurements per animal: 3 sections per animal*20 measurements per section). For immunohistochemical staining of colonic proliferating cells, 5 µm sections (from 8 animals per treatment per time-point) were deparaffinized, rehydrated and treated for antigen retrieval in citrate buffer (pH 6.0) at 95°C for 20 min, followed by cooling in tris-buffered saline and tween 20 (TBSt) buffer and blocking with 10% normal goat serum (Invitrogen™). To detect proliferating cells, sections were incubated with primary antibody (anti-PCNA antibody, PC10 mouse anti-rat IgG2a monoclonal antibody, Merck-millipore, Darmstadt, Germany, MAB424R; 1:500) and secondary antibody (Goat anti-Mouse IgG (H+L) Superclonal™ Secondary Antibody, Alexa Fluor^®^ 555, ThermoFisher Scientific, Waltham, Massachusetts, USA; 1:300). Nuclei were stained with Hoechst 33342 Solution (Invitrogen, ThermoFisher; 1:1000 dilution). 10 high quality 16bit grayscale images were captured per animal at 20X magnification (80 representative images/treatment) using Leica DM6b microscope fitted with appropriate fluorescence filters along with their corresponding nuclei images.

Image analysis was done using a semi-automated in-house workflow that we described previously (58), encompassing image data-extraction by Cell Profiler 3.1.8 (Broad Institute, Cambridge Massachusetts USA; www.cellprofiler.org) and analysis in FCS Express 6 Flow plus Image (*De Novo* Software, CA, USA, www.denovosoftware.com). The relative number of proliferating cells was obtained by normalising with the total number of Hoechst positive nuclei (PCNA : Hoescht ratio) in each image.

### Other statistical analysis

Normality of data (Shapiro-Wilk test) and statistical differences were checked with a limit of significance set at *P* < 0.05 in GraphPad Software 8.1.1. Comparison of the squared Bray Curtis distance, histological morphometric measurements and proliferating cells between treatments were performed by Mann Whitney U-test (non-parametric) or t-test (parametric), whereas comparison among different time-points (histological morphometric measurements and proliferating cells) were assessed by one-way ANOVA (parametric) or Kruskal-Wallis test (non-parametric) using a Dunnett’s test for multiple comparisons.

## Results

### Pre-weaning fibrous diet accelerates gut microbiota maturation in early-fed piglets

We have previously shown that pre-weaning consumption of fibrous feed accelerates the development of the intestinal microbiota in suckling piglets (42), which is underpinned by a pre-weaning microbiome that is more adapted towards a typical post-weaning microbiota composition. As the present study employed a similar intervention design, our initial analyses were geared to confirm the anticipated impacts of early feeding with fibrous feed on the colonisation pattern and maturation of the gut microbiota.

**Figure 2:**
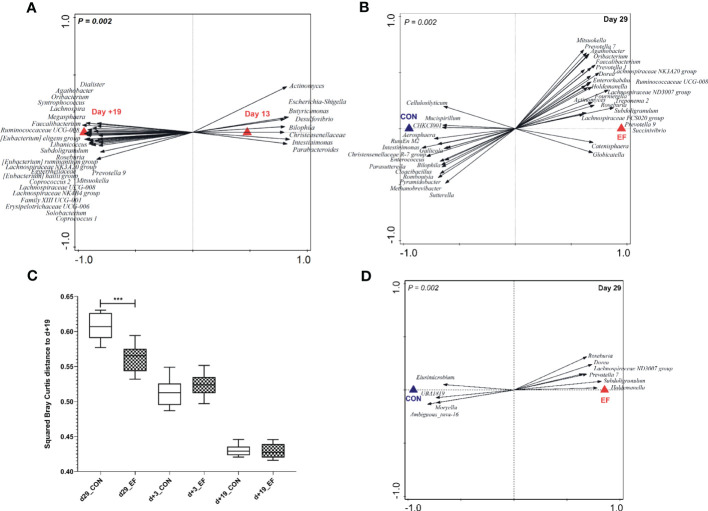
Pre-and post-weaning microbiota composition (irrespective of treatment) and pre-weaning acceleration in the early-fed (EF) group as compared to the control (CON) group (genus level). **(A)** Redundancy analysis of pre- (d13) and post-weaning (d+19) time-points (explained variation = 41.81%; *P = 0.002*) with associated microbial groups. Microbial groups visualized have a minimum response score of > 0.80 on horizontal axis. **(B)** Redundancy analysis (colon, d29) of the EF and CON group (explained variation = 16.1%; *P* = 0.002) with discriminating microbial groups (response score > 0.60). **(C)** Comparison of squared Bray Curtis index (distance to d+19 time-point) over time, between the two groups. **(D)** Redundancy analysis (jejunum, d29) of the CON and EF group (explained variation = 11.6%; *P* = 0.002) with discriminating microbial groups (response score > 0.65) at genus level. Significant differences between groups were assessed by student t tests or Mann-Whitney U tests (***: *P* < 0.001).

The typical pre- and post-weaning associated microbiota compositions were analysed and compared using rectal swabs obtained at 13 days of age to reflect the pre-weaning microbiota (preceding substantial eating behaviour of the fibrous feed) and 19 days post-weaning (day+19) as a reflection of the typical post-weaning microbiota (42). Specific microbial groups were associated with the pre- and post-weaning stages irrespective of the early feeding treatment (**Figure 2A**), which was corroborated by analysis of the individual time-points day13 and day+19 that did not reveal any treatment-associated differences (**Supplementary Figures 2A, B**). Notably, the microbial groups that were most discriminant between the pre-weaning (day 13) and post-weaning (day+19) microbiota composition strongly resembled those found in our previous study (42), which is exemplified by more than 50% overlap in the microbial genera identified (**Supplementary Table 4**).

Colon microbiota analysis of sacrificed piglets at weaning (29 days of age) revealed that early-fed (EF) piglets had significantly expanded microbial genera (**Figure 2B**; **Supplementary Figure 2C**; **Supplementary Table 5**) that are typically associated with the post-weaning microbiota (**Figure 2A**), including *Prevotella, Subdoligranulum, Faecalibacterium, Roseburia* and *Megasphaera*. Conversely, colon samples obtained from control (CON) piglets appeared to be enriched in microbes belonging to *Enterococcus*, *Intestimonas, Christensenellaceae R-7 group, Romboutsia*, *Methanobrevibacter* (**Figure 2B**; **Supplementary Figure 2**; **Supplementary Table 5**) which are associated with the pre-weaning microbiome (**Figure 2A**). Besides evaluating “accelerated-maturation” at weaning, we also assessed the “persistence of the impact of early feeding” 3 days post-weaning (day+3). We observed that the pre-weaning microbiota differences in the colon (day29) persisted in a modest way to the first time-point analysed after weaning (day+3), which is reflected by the higher relative abundance of the genera *Coprococcus, Marvinbryantia, Selenomonas, Prevotella, Prevotellaceae NK3B31 group, Eubacterium xylanophilum, Catenisphaera* and *Agathobacter* in the colonic microbiota of EF piglets versus *Peptostreptococcus* and *uncultured-39* found in the CON group (**Supplementary Figure 2D**; **Supplementary Table 5**). In contrast, and according to our anticipation, the pre-weaning treatment effects on the colonic microbiota were no longer detectable at the later post-weaning time-point (day+19) (**Supplementary Figure 2B**). The accelerated microbiome development in EF piglets was further underpinned by the significantly lower Bray Curtis distance of the EF samples at 29 days of age, relative to the ‘mature’ post-weaning time-point (day+19) (**Figure 2C**), where microbiota convergence was observed.

In the early life intervention study presented here, we also detected a significant effect of early feeding on the jejunal microbiota on day29 (**Figure 2D**), which contrasts with a previous study using a similar design (58). Remarkably, the distinctive microbial groups were previously identified to reflect the early feeding intervention in the colon microbiota (i.e., EF-associated) including the increased relative abundance of *Roseburia, Lachnospiraceae ND3007 group, Prevotella, Subdoligranulum, Holdemanella, Dorea* (**Supplementary Table 6**). Notably, the jejunal microbiota differences elicited by early feeding were no longer detectable post-weaning (day+3), which could reflect a more modest impact of early feeding observed in this intestinal region. Although the impact of early feeding on the jejunal microbiota is moderate compared to the colon microbiota, these findings are suggestive of a similar influence of early feeding on small and large intestinal locations, although with a varying degree of strength.

### Early feeding leads to “accelerated maturation” in intestinal mucosal transcriptome

To explore the consequences of early feeding on the time-resolved mucosal transcriptional program, we conducted transcriptome analyses (in jejunal and colonic tissue) on the day of weaning (day29), and two time-points post weaning (day+3 and day+21). At each time-point 10 animals from both groups (CON and EF) were sacrificed to harvest jejunal and colonic tissue samples, which were processed for different purposes (see M&M for details, and see below), including tissue transcriptome analysis using RNA sequencing on an Illumina Hiseq platform, generating an average of 25 million PE reads per sample.

**Figure 3:**
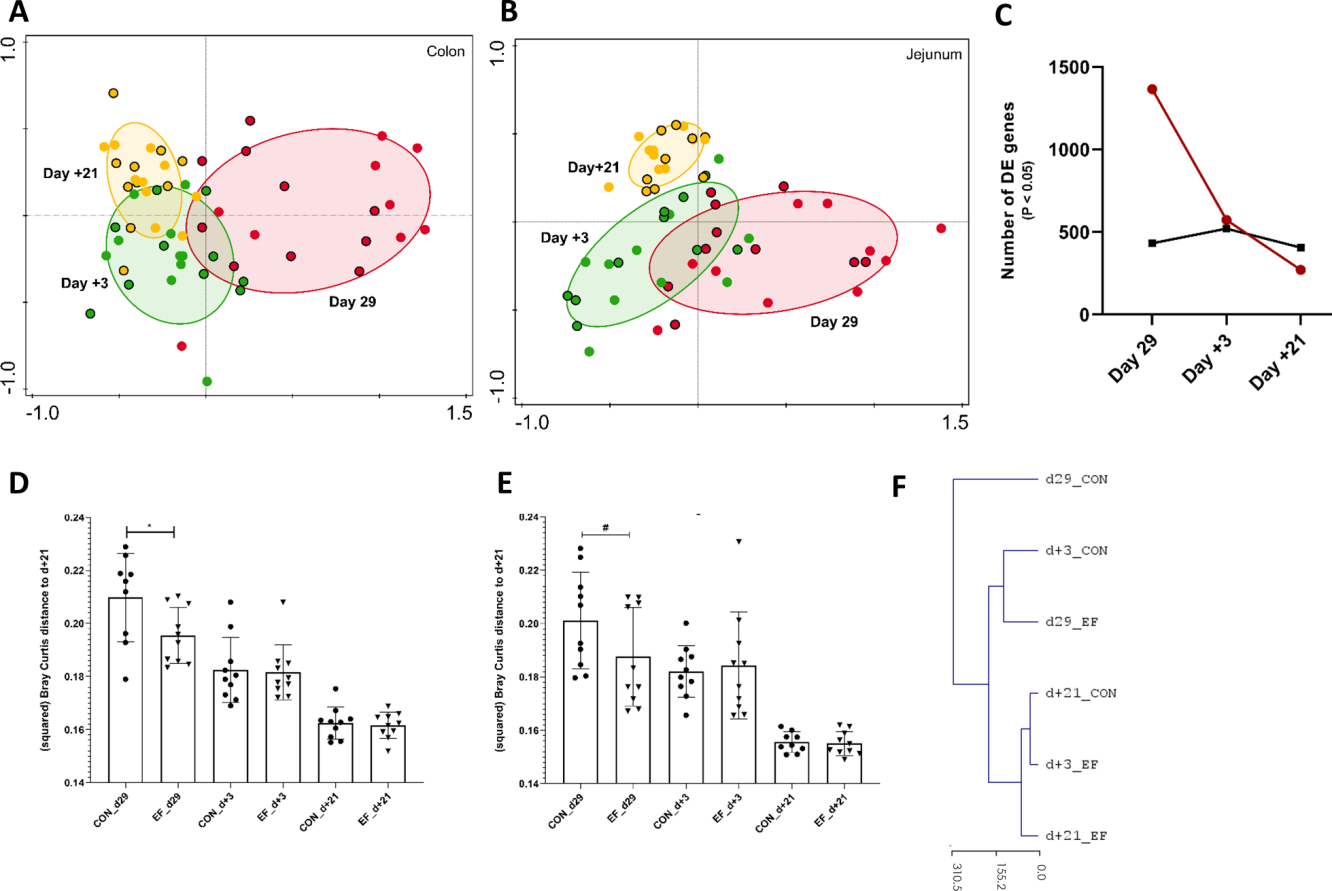
Jejunum and Colon transcriptome alterations over time (pre- and post-weaning). **(A)** Principal component analysis of whole colon transcriptome (PC1 = 26.6%, PC2 = 10.4%) at day29 (pre-weaning; red), day+3 (post-weaning; green) and day+21 (post-weaning; yellow) denoted by circles with (Early-fed or EF piglets) and without (control or CON piglets) black border. **(B)** Principal component analysis of whole jejunal transcriptomics (PC1 = 21.1%, PC2 = 11.6%) at day29, day+3 and day+21. **(C)** Number of differentially expressed genes (DEG; EdgeR test: *P* value < 0.05) at day29, day+3 and day+21 in colon (red) and jejunal (black) samples. **(D)** CON and EF group comparison of squared Bray Curtis distance at day+21 time-point in **(D)** colon and **(E)** jejunum. **(F)** Unsupervised hierarchical clustering (euclidean distance) of whole colon transcriptome using normalised (logCPM) expression values (averaged per group per time-point) and scaled by the mean value of day+21 gene expression (irrespective of treatment). *: *P* < 0.05; #: *P* < 0.1.

Global comparison of both colonic and jejunal transcriptome profiles by principal component analysis (PCA; **Figures 3A, B**) revealed a clustering of samples based on age, irrespective of the pre-weaning treatment (EF versus CON). To investigate the impact of early feeding on the jejunal and colonic transcriptome, differentially expressed genes (DEG; EdgeR test) were assessed at each time-point separately. These analyses revealed that day29 has the most DEG in colon tissue, and these differences between treatment groups decrease over time or “converge” post-weaning (**Figure 3C**). The number of DEG (*P* < 0.05) decreased from 1366 genes (at day29) to 573 (at day+3) and 270 (at day+21) genes, respectively, however, the number of differential genes post-weaning became negligible when the *P* value was corrected for multiple testing (False discovery rate or FDR < 0.1; **Supplementary Figure 1B**). Much less DEG were observed in jejunal tissue (compared to colon), and the number of DEG remained more or less similar over time (*P* < 0.05; **Figure 3C**), although FDR correction (**Supplementary Figure 1B**) somewhat refined this conclusion, demonstrating a declining number of DEG also in the jejunal transcriptome, analogous to the colon. To evaluate the development of gene expression over time, we compared the treatment groups in relation to the last post-weaning time-point (day+21), where we observed the convergence of the transcriptome data in EF and CON groups.

Strikingly, at day29 the colon transcriptome profile of the EF group had a significantly smaller distance (Bray Curtis) to the “convergent transcriptome average of both groups” (day+21) compared to the CON group (**Figure 3D**). Similarly, the day29 jejunal transcriptome profile of the EF group tended to resemble the day+21 “convergent jejunal transcriptome of both groups” more closely as compared to the CON group (*P* = 0.06, **Figure 3E**). These observations were also evident in PCA analyses (**Figures 3A, B**) that illustrate the EF day29 transcriptomes (red circles with black border) clustering in closer proximity to the day+21 transcriptome profiles relative to the CON day29 transcriptome (red circles). This was further corroborated by principal response curves (PRC) analysis which assessed the temporal effects of the EF treatment and detected the biggest impact of early feeding at day29 in the colon (5.4% of total variation; 499 permutations; *P* = 0.01), as well as by unsupervised hierarchical clustering of the colon transcriptome, positioning the day29 CON group transcriptome farthest from the post-weaning time-point and highlighting its closer resemblance to the day29 EF group transcriptome (**Figure 3F**). Remarkably, the EF group transcriptome determined on day+3 appeared to be more similar to the average day+21 transcriptome profile, as compared to the CON transcriptome. Unlike colon, we did not detect a significant impact of early feeding over time in jejunum (PRC; 4.8% of total variation; *P* = 0.12), thereby reflecting the relatively moderate impact of early feeding on the jejunum. The unsupervised hierarchical clustering of the jejunal transcriptome positioned the day+3 CON farther from the post-weaning (day+21) time-point compared to the day+3 EF (**Supplementary Figure 3A**), which might indicate the impact of EF on jejunum more relevant at day+3. Overall, these results illustrate that the largest effect of early feeding is detected at the end of the pre-weaning stage (day29), and that this effect rapidly diminishes after weaning to converge to indistinguishable transcriptome profiles three weeks post-weaning (day+21). Moreover, this also reveals that early feeding appears to induce an acceleration of the molecular development of the intestine mucosa, particularly in the colon mucosa.

### Functional analysis of the DEG due to early feeding

To further explore the transcriptional response due to early feeding, pathway analysis was performed on the DEG at individual time-points. Analogous to the larger number of DEG identified in the colon transcriptome at day29, more up- and down-regulated canonical pathways were significantly enriched at day29 (32 canonical pathways) compared to the post-weaning time-points day+3 (10 canonical pathways) and day+21 where no pathways appeared to be enriched (**Supplementary Figure 3B**). These analyses re-affirmed the convergence of the colon transcriptome within 3 weeks post-weaning in the treatment groups. On the other hand, reflective of the lower and more consistent numbers of DEG identified in the comparison of the jejunal transcriptomes, pathway analysis at all three time-points (day29, day+3, day+21) identified only 3 and 7 significantly enriched canonical pathways (in EF as compared with CON piglets) at day29 and day+3, respectively (**Supplementary Figure 3C**).

**Figure 4:**
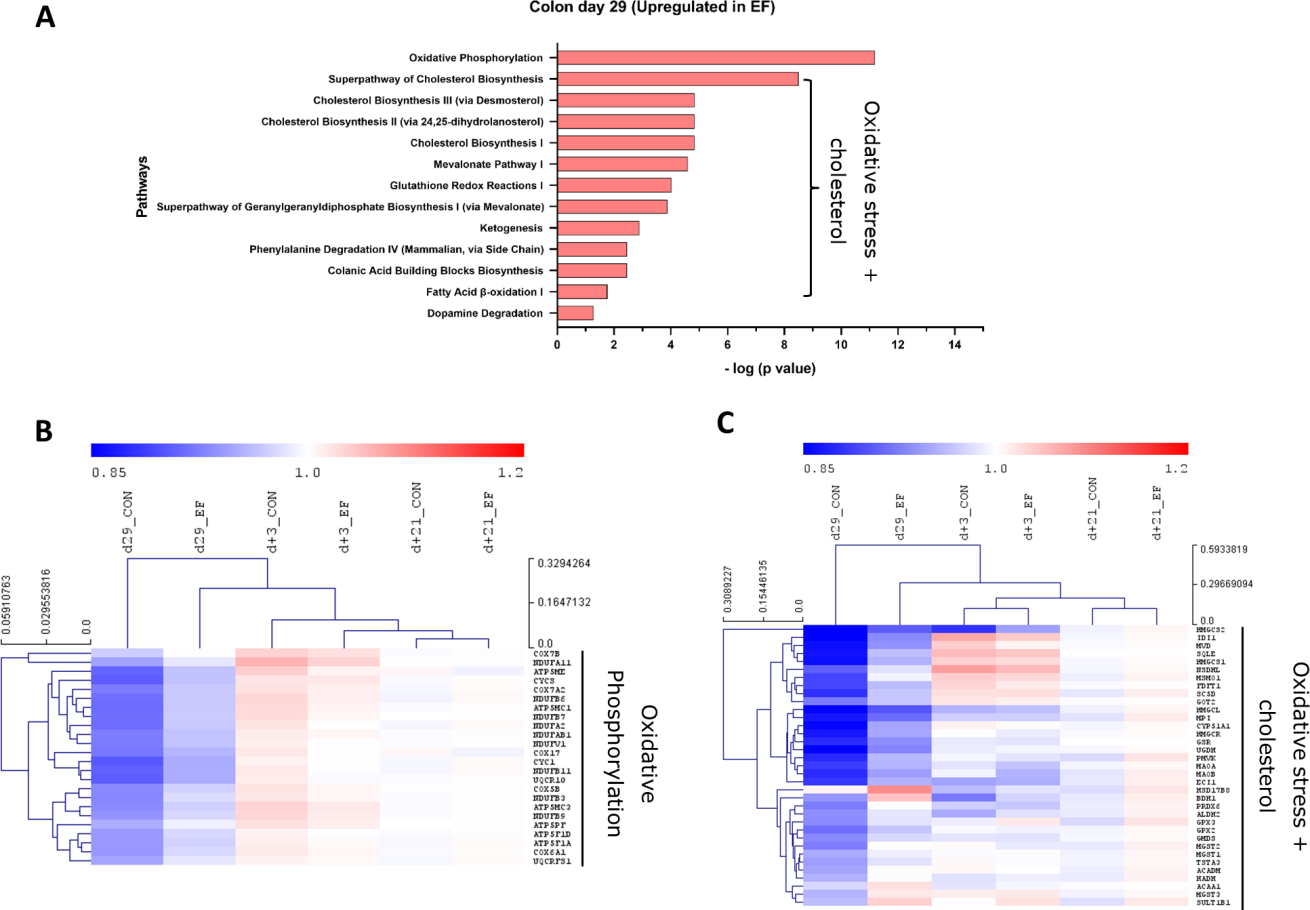
Pathway analysis (upregulated) in colon mucosa. **(A)** Canonical pathways upregulated in the early-fed (EF) group compared to the control (CON) group at day29. Identified in Ingenuity pathway analysis (IPA) having logP value ≥ 1.3 (enrichment score from Fisher’s exact test) and an absolute Z score ≥ 2 (assessing the match of observed and predicted up/down regulation patterns). Hierarchical clustering of expression profiles over time for **(B)** Oxidative phosphorylation pathway genes **(C)** Oxidative stress + cholesterol related pathway genes including pathways including glutathione redox, ketogenesis, dopamine degradation. The normalised expression values (averaged per group per time-point) are scaled by the mean value of total day+21 expression (irrespective of treatment).

**Figure 5:**
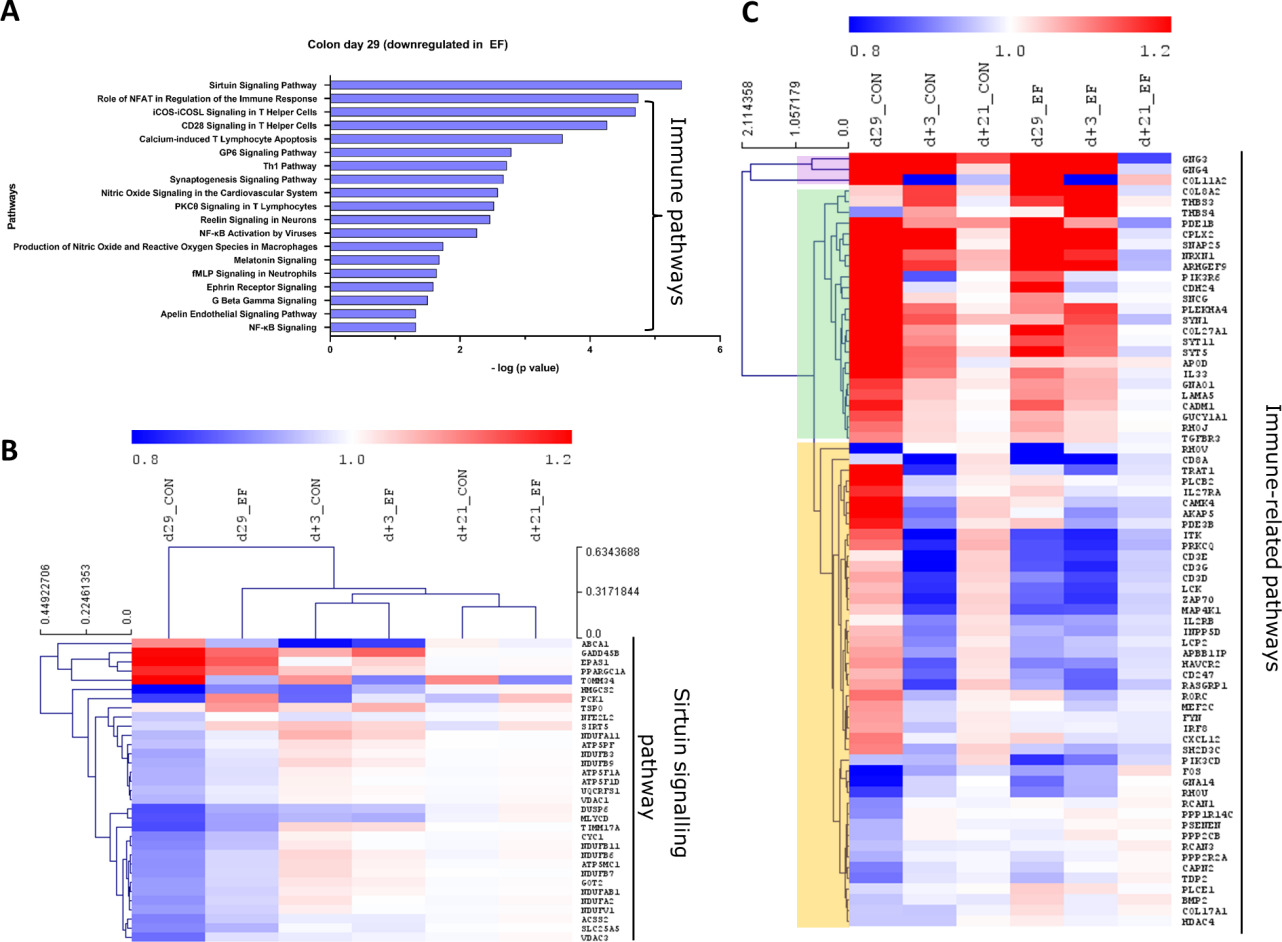
Pathway analysis (downregulated) in colon mucosa at 29 days of age. **(A)** Canonical pathways downregulated in the early-fed (EF) group compared to the control (CON) group. Identified in Ingenuity pathway analysis (IPA) having logP value ≥ 1.3 (enrichment score from Fisher’s exact test) and an absolute Z score ≥ 2 (assessing the match of observed and predicted up/down regulation patterns). **(B)** Hierarchical clustering of expression profiles over time for sirtuin signalling pathway genes. **(C)** Heatmap showing immune pathway genes (without hierarchical clustering). The normalised expression values (averaged per group per time-point) are scaled by the mean value of total day+21 expression (irrespective of treatment).

In the EF group, oxidative phosphorylation and cholesterol biosynthesis pathways were significantly upregulated in the colon mucosa at day29 (**Figure 4A**). In addition, oxidative stress related pathways such as glutathione redox reactions, ketogenesis and fatty acid ß oxidation pathways were enriched in EF compared to CON piglets. On the other hand, the significantly downregulated pathways in EF group were sirtuin signalling and immune response pathways (**Figure 5A**), such as CD28 signalling in T Helper cells, calcium induced T Lymphocyte Apoptosis, GP6 signalling, Th1 pathway and NFκB signalling among others. Three days after weaning (day+3), the colon transcriptome in EF piglets displayed upregulation of pathways such as Integrin, Leukocyte extravasation, p38 MAPK, Netrin, TREM1 signalling, associated with extra-cellular matrix (ECM), immune response and external (stimulus) stress response (**Supplementary Figure 3B**), which could reflect the tissue’s adaptive response to “weaning-associated stress”. Notably, the persistence of pre-weaning altered pathways was not observed post-weaning in colon transcriptome of EF piglets. However, jejunum showed persistence in the upregulation of oxidative phosphorylation pathway at both day29 and day+3 (**Supplementary Figure 3C**), although modest numbers of genes were involved.

To investigate the evolution of gene expression in EF and CON groups over time, unsupervised hierarchical clustering was performed using the “point of convergence” (averaged day+21 expression values for each gene; irrespective of prior treatment) to scale and compare the transcriptome profiles over time. Time-resolved (colonic) gene expression patterns related to oxidative phosphorylation and oxidative stress-related pathways (including cholesterol pathway) displayed clear convergence of the gene expression towards day+21 (**Figures 4B, C**). In addition, the EF group (day29) was substantially closer to the post-weaning “convergent” time-point (day+21), compared to the CON group at the same time-point (**Figures 4B, C**), confirming an accelerated activation of these pathways due to early feeding. Sirtuin signalling associated genes displayed similar behaviour as we observed with the upregulated pathway genes, with similar expression as the post-weaning time-point (day+21) (**Figure 5B**), thus affirming the acceleration of the gene expression program due to early feeding. Notably, the hierarchical clustering of the DEG associated with immune system functions appeared predominantly age-driven (**Supplementary Figure 4A**). However, this age-dependent gene expression conclusion was dictated by a subset of the immune system associated genes (purple and green clusters in **Figure 5C**). On the other hand, the other subset of immune system associated genes (yellow cluster in **Figure 5C**) did display the typical accelerated evolution of expression in EF piglets when analysed separately (**Supplementary Figure 4B**), as previously seen in other pathways. Overall, we detect a mixed observation in immune-related genes where the upper panel of genes displays age-related programs, and the lower panel displays acceleration by the early feeding treatment.

The leading genes of day+3 in colon showed a similar acceleration pattern in the EF group as observed previously, with both day29 and day+3 clustering with the convergent day+21 transcriptome (**Supplementary Figure 4C**). Of note, only one gene, GADD45B (part of sirtuin pathway; involved in cell cycle regulation) was found to be common on both day29 and day+3, although not significant at day29 and with an inverse fold change at day+3 (*P* = 0.01). Though in jejunum much fewer altered pathways were detected in EF as compared to the CON group, hierarchical clustering of those pathway identified genes at day29 and day+3 also support the accelerated (pre-weaning) maturation in the EF group (**Supplementary Figures 4D, E**).

**Figure 6:**
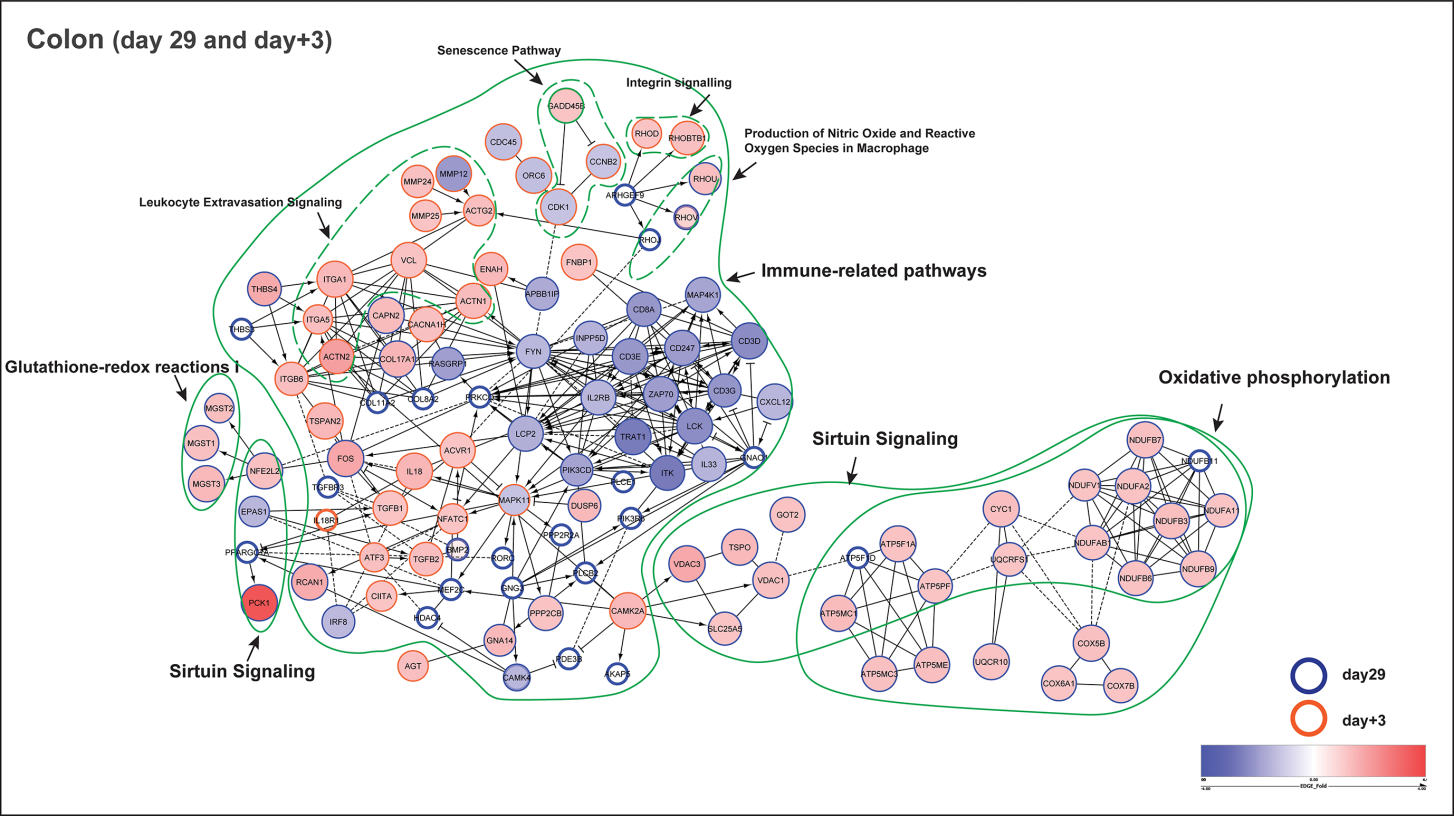
Functional network of pathway identified genes (colon) at day29 and day+3. Functional protein-protein interaction of up- and down-regulated genes in early-fed (EF) compared to control (CON) group. The circles or nodes represent genes and edges represent interactions between genes, as determined by Reactome. Arrows represent directed interactions, bar-headed arrows indicate inhibition reactions and dotted lines indicate predicted relationships. Pathway identified genes from time-points day29 (blue bordered) and day+3 (orange bordered) are shown in this figure. The upregulated (red nodes) and down-regulated (blue nodes) genes are represented by their fold change (EdgeR test) in a blue-red gradient scale, where the size of the node is proportional to their significance (*P* value; EdgeR test). The genes having *P* value > 0.1 are depicted as white nodes. The dotted green lines depict different pathways associated with those genes. Of note, the gene GADD45B (part of senescence and sirtuin pathway) was observed at both day29 (fold change = - 1.3; *P* = 0.13) and day+3 (fold change = 1.2; *P* = 0.01).

Additionally, protein-protein interaction networks were generated using the pathway associated leading gene in both day29 and day+3 (Cytoscape, Reactome FIviz plugin). From the colon transcriptome, a total of 180 pathway identified genes combining day29 (142 genes) and day+3 (38 genes) were used to construct the interaction network (**Figure 6**). In the network, the most prominent gene clusters were associated with oxidative phosphorylation, immune-related and sirtuin signalling pathways. Both oxidative phosphorylation and sirtuin signalling pathways are known to be active in mitochondria for redox metabolism, having common genes observed in the network such as ATP5F1A, ATP5F1D, ATP5MC1, ATP5PF (mitochondrial ATP synthase subunits), CYC1 (cytochrome bc1 complex subunit), NDUFA2, NDUFA11, NDUFB3, NDUFB6, NDUFB7, NDUFB9, NDUFB11, NDUFAB1, NDUFV1 (NADH:ubiquinone oxidoreductase subunits) and UQCRFS1, that encode key enzyme complexes in the electron transport chain reaction. Besides, sirtuin signalling is also involved in homeostasis and cellular adaptive response to external environmental stimuli, which can be observed in the altered transcription-regulating gene expression of PPARGC1A, NFE2L2 and EPAS1 (**Figure 6**), indicating a link between external stimuli and the regulation of cellular metabolism in the colon. Furthermore, PPARγ was identified as one of the potential upstream transcriptional regulators for the mucosal gene expression response to fibrous feed (Activation Z score = 3.4; Upstream Regulator Analysis, IPA). The other important large gene cluster in the network represents immune-related pathways, including tyrosine kinase family hub genes such as FYN, LCK, that are involved in cell growth and immune activation, chemokine receptors (CXCL12), as well as T cell development (proliferation, differentiation) and signal transduction (antigen recognition) genes like LCP2, ZAP70, ITK, TRAT1, CD3D, CD3E, CD3G, CD247, IL33 and IL2RB (**Figure 6**). Interestingly, this cluster of immune genes were previously observed to be deviating in the CON group at day+3, displaying an abrupt change of gene progression from day29 to day+21 (**Figure 5C**), which was not observed in the EF group.

In the functional network, we clearly observed the immune system to be more responsive in the EF piglets at day+3 potentially due to the newly emerging post-weaning situation (**Figure 6**). This is reflected by the activation of inflammation-related genes such as IL18, IL1RL1, TGFB1,TGFB2; leukocyte extravasation (i.e., leukocyte migration from peripheral blood into tissues site of inflammation) pathway identified genes including MMP12, MMP24, MMP25, as well as ECM (Integrin and Paxillin signalling; ACTG2, ACTN1, ACTN2, ITGA1, ITGA5, RHOD, RHOBTB1, VCL) genes that regulate cell migration and intercellular communication during inflammation/stress. MAPK11 can be one of the hub genes or central regulator of the above mentioned biological processes, as observed in the functional network. Notably, GADD45B gene (part of senescence and sirtuin pathway) was found at both day29 and day+3, with significant upregulation (fold change = 1.2; *P* = 0.01) after weaning (day+3), and is known to arrest cell growth and proliferation *via* blocking cell cycle proteins encoded by CDK1 and CCNB2, as seen in the network (**Figure 6**).

Even though jejunum mucosal transcriptomes only displayed a modest impact of early feeding (as described before), we investigated the functional interaction in the jejunum using a total of 43 pathway identified genes combining day29 (13 genes) and day+3 (32 genes) to build the interaction network (**Supplementary Figure 5**). As anticipated, a substantially smaller functional network was observed, mostly revealing interactions between genes involved in oxidative phosphorylation and sirtuin signalling pathways, and some smaller networks associated with immune system functions. Notably, genes belonging to oxidative phosphorylation such as ATP5MC1, ATP5MC3, ATP5ME, COX17, COX7A2, NDUFA2 were found in both jejunum and colon at day29, whereas only one gene RHOD (Rho-related GTP-binding protein; involved in reorganization of the actin cytoskeleton and membrane transport) was found to be common between jejunum (part of ILK signalling) and colon (part of Integrin signalling) at day+3. Taken together, these findings indicate that EF piglets show an “alerted system” that displays an enhanced responsiveness to external stimuli of feed and microbiome development in colon and moderately in the jejunum, compared to the CON group.

### Effect of early feeding on mucosal morphometry and proliferative cells at pre- and post-weaning period

**Figure 7:**
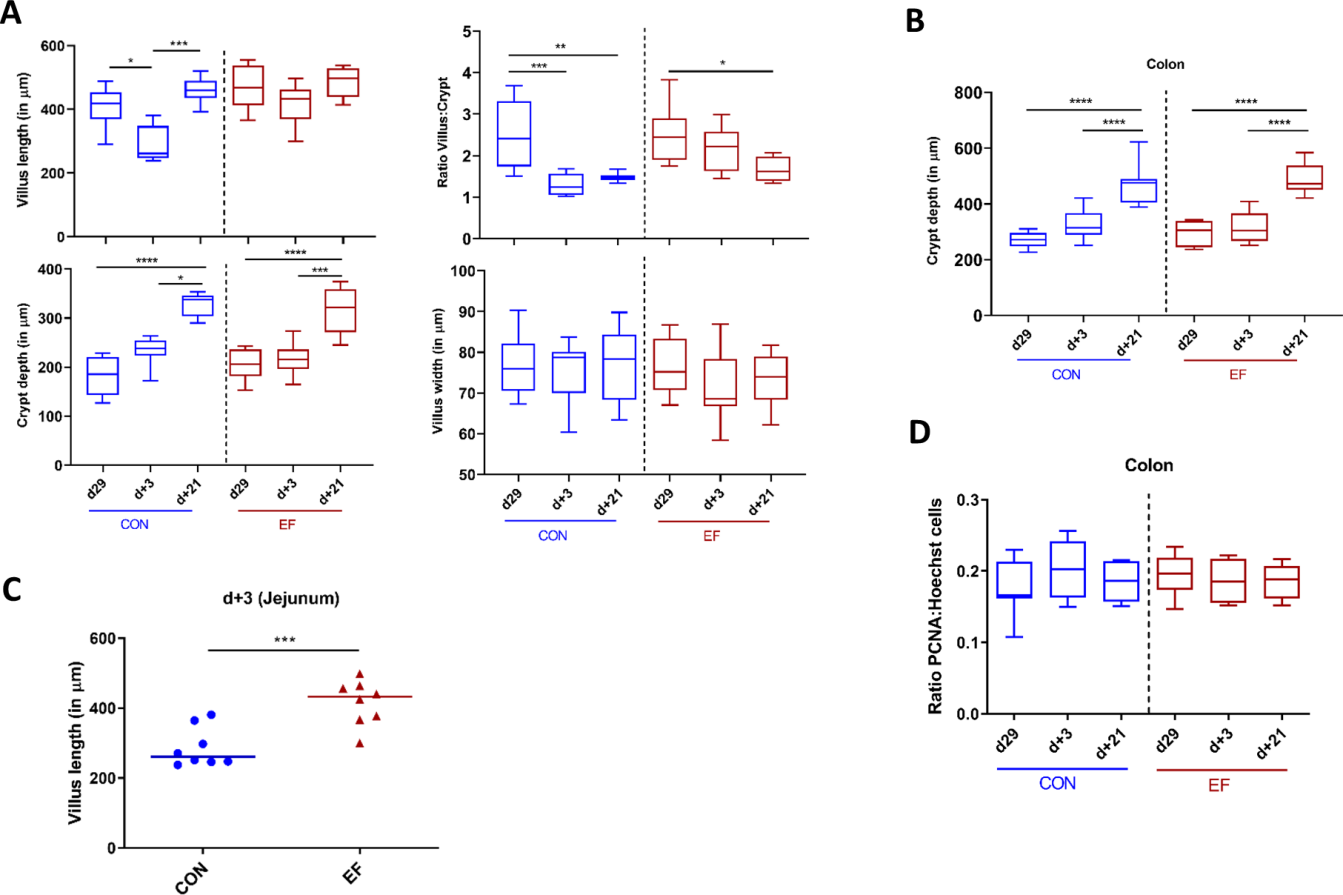
Intestinal morphometric analysis at day29, day+3 and day+21 time-points in jejunum and colon. **(A)** Measurements in jejunal tissue: Villus length, crypt depth, calculated villus:crypt (V:C) ratio and villus width. **(B)** Crypt depth measurement in colon tissue. **(C)** Comparison of villus length between early-fed (EF) and control (CON) group at day+3. **(D)** Colonic epithelial proliferating cells estimated by semi-quantitative image analysis by calculating the ratio of PCNA positive: Hoechst nuclei stain. Differences between groups were assessed by either Mann-Whitney U test or Kruskal Wallis test (non-parametric). (*: P < 0.05; **: P < 0.01; ***: P < 0.001; ****: P < 0.0001).

In this study, we also investigated the intestinal mucosal changes (over time) in CON and EF piglets by evaluating their mucosal morphometry. Jejunum and colon samples (n = 8 per group per time-point per location) were assessed using H&E stained tissue images obtained at day29, day+3 and day+21. These analyses revealed clear age-related morphometric development of the jejunum mucosa, which was especially apparent in increasing crypt depth over time, while the villus length and width were not significantly different between the weaning day (day29) and three weeks post weaning (day+21). This observation is paralleled by a crypt depth dependent decrease in villus:crypt ratio over time (**Figure 7A**). Analogously, in colonic mucosa the crypt depth also increased over time, which was especially clear at day+21 (**Figure 7B**). These differences between day29 and day+21 were observed in both groups of piglets (EF and CON).

Comparative analysis between the CON and EF group was performed for each morphometric measurement. Remarkably, at day+3 we observed significantly higher villus length in EF compared to CON piglets (**Figure 7C**). In other words, EF (unlike CON) piglets, did not show a drastic change in villus length over time, indicating smoother weaning transition. This was also reflected in the significant difference between day29/+21 and day+3 within the CON group, suggesting an impact of weaning on jejunal mucosa in the CON group (**Figure 7A**). However, no other morphometric differences in crypt depth, ratio of villus:crypt, villus width (jejunum) and crypt depth (colon) were observed between the treatment groups (**Supplementary Figures 6A–C**).

Next, we evaluated the potential alterations in epithelial proliferation (due to increasing crypt depth), and subsequently assessed the effect of early feeding. The number of epithelial proliferating cells were estimated in colon mucosa (n = 8 per group per time-point) by employing the previously standardised IHC quantification protocol (58). Previous studies suggested that increased crypt depth is correlated with increased epithelial proliferation rates in the mucosa (59), however, we did not observe a reflection of increasing crypt depth over time in the number of proliferating cells, which remained stable over time in both CON and EF (**Figure 7D**). Further, no difference was detected in the number of proliferating cells (ratio PCNA: Hoechst nuclear stain) between the CON and EF groups at any time-point (**Supplementary Figure 6D**), which was further supported by the lack of (significant) changes in the PCNA gene expression level (**Supplementary Figure 7**). This is contrasting to our previous study where increased number of proliferating cells were found in EF group at weaning (58). Additional measurements of (specific) differentiated cell type-specific markers could refine this view.

## Discussion

Dietary fibres are generally known to influence intestinal health, *via* unexplored biological mechanisms that have been proposed as a consequence of microbial fermentation into short chain fatty acids (SCFAs). In this study, we have confirmed the impact of early feeding (fibrous feed including dietary fibres like GOS, inulin, resistant starch) on colon microbiota, with accelerated microbiota maturation in suckling piglets, as also observed in a prior study employing a similar design (42). The accelerated maturation in the early-fed (EF) piglets as compared with control (CON) piglets, was illustrated by the closer resemblance of pre-weaning microbiome with the post-weaning microbiota composition, with an expanded microbiota at day29 comprising of typical post-weaning microbial groups *like Prevotella, Subdoligranulum, Faecalibacterium, Roseburia* and *Megasphaera.* Moreover, we could additionally evaluate the persistence of EF-associated microbes post-wearing and found persistence to some extent at three days post weaning (day+3) but not after three weeks (d+19). The similar microbiota composition in CON and EF piglets after weaning indicates the rapid microbiota adaptation due to diet and supports the previously observed “convergence” of microbiota post-weaning. Contrary to our previous findings (58), we observed a significant impact of early feeding on jejunal microbiota at day29, which did not persist post-weaning at day+3. This can be possibly explained by the lower pre-weaning feed intake in the previous study (58) that may have been compromised by a diarrhoeic episode during the third week of age, thereby potentially weakening the EF impact. This is because we observed similar microbial signature in jejunum, with microbial groups like *Subdoligranulum*, *Coprococcus* tending to correlate with the individual eating scores of the piglets (58) (data not shown), suggestive of the impact being driven by the amount of the feed consumed.

The primary aim of the current study was to investigate whether early feeding (pre-weaning provision of fibrous feed) can modulate the host mucosal transcriptome over time in colon and jejunum tissues. To this end, we determined mucosal tissue transcriptomes to capture the overall tissue responses. This approach has the advantage that sub-epithelial responses (e.g., those occurring in immune cells in the lamina propria) are included, and thereby a more holistic tissue response analysis is enabled. However, this approach comes with a trade-off because cell-lineage specific responses may be diluted and less prominently observed. This can in part be overcome by analysis of cell lineage specific marker genes within the transcriptome (**Supplemental Figure 7**) to reveal cell-composition differences elicited by the intervention. Moreover, to refine these tissue transcriptomes subsequent analyses could focus on single cell transcriptome sequencing approaches and/or immunohistochemistry or *in situ* hybridization. The tissue transcriptomes revealed that early feeding accelerates not only the luminal microbiota (see above) but also the host transcriptome maturation in neonatal piglets, in addition to impacting the jejunal morphometry during the weaning transition. Intriguingly, the results demonstrate the coinciding timeframe of “accelerated maturation” as well as convergence after weaning both in the intestinal microbiota and transcriptome profile, indicating their potential interrelation. Maximum impact of early feeding was detected at weaning (day29), which was followed by convergence of transcriptome three weeks post-weaning (day+21). The “convergent” post-weaning time-point (day+21) was exploited to evaluate the maturation of the piglets at weaning and few days after weaning. It is to be noted that this study comprised of mixed effects of weaning-induced, age-related as well as treatment-associated transcriptome changes. Strikingly, the EF treatment effect seemed to overrule the aging/weaning effect, as was clearly evident from the hierarchical clustering of transcriptome profiles, especially in colon and much modestly in jejunum. This is consistent with literature (60–63) as the microbial fermentation of fibres leading to SCFA production, predominantly occurs in the distal part of the gastrointestinal tract, thus a larger effect on colon mucosa due to fibre consumption is likely. However, separating the impact of the microbes from their metabolic products remains difficult. The key question is whether early feeding is causative for microbiome/transcriptome changes. We have previously established the association of eating and microbiome changes (42), however the causative relation with mucosal transcriptome changes is difficult to determine in this study, although it seems likely that “feed in combination with microbiome” drives the predominant transcriptome changes (larger than age-effect).

SCFAs (particularly butyrate) form an important energy source for the intestinal epithelium and subsequently influences the epithelial cell homeostasis (64–66). Considering that SCFAs are fatty acids that are transported into colonocytes and eventually oxidised in mitochondria (66, 67), it is reasonable that the pathways involved in cellular energy or mitochondrial metabolism, are altered in the EF group (higher levels of colonic SCFAs shown in a study (58) with similar experimental design), which were enriched in SCFA producing microbes (*Prevotella, Subdoligranulum, Faecalibacterium, Roseburia* and *Megasphaera*). This was further supported by the increased expression levels of SCFA transporters like SLC16A1, SLC26A3 (**Supplementary Figure 8**) in the EF piglets at weaning, indicating that the CON piglets are less well-prepared to tackle the (post-weaning) changing circumstances (feed, microbiome, SCFA). At day29, EF piglets displayed an upregulation of genes associated with pathways including oxidative phosphorylation, cholesterol biosynthesis, glutathione redox reactions, ketogenesis and fatty acid ß oxidation (related to cellular metabolism and oxidative stress) and a downregulation of sirtuin signalling and immune-related pathways. This is consistent with previous studies which supplemented animal feed with non-digestible carbohydrates, and has shown to activate fatty acid oxidation in different animal models, including pigs (68), rats (40). Moreover, a germ free versus conventionally raised mice study (66) has previously shown the impact of microbiota on energy homeostasis, specifically butyrate promoting oxidative metabolism in colonocytes by upregulating genes involved in fatty acid metabolism, glycerolipid metabolism, TCA cycle and mitochondrial oxidative phosphorylation (OXPHOS). The results presented here thus are in agreement with these studies, although our study highlights that these processes can be initiated during early life by providing piglets with fibrous feed during lactation, whereby the transition to solid feed at weaning can be smoothened and does not require the mucosa to adjust abruptly to these novel feed components.

Our analysis identified PPARγ as an upstream transcriptional regulator that mediates the effects of the dietary fibres on gene expression, which is in accordance with a previous study (36) that evaluated different fibre sources in mice. Furthermore, EF piglets displayed an increased expression of oxidative stress pathways in colon mucosa. This is in line with previous knowledge that enterocytes contacted by enteric commensal bacteria (and/or their products) are known to stimulate transient oxidative stress generating physiological levels of reactive oxygen species (ROS) (69, 70). Microbial-elicited ROS may serve as second messengers to mediate cellular proliferation, motility and modulate innate immune signalling which is necessary for commensal-induced gut epithelial homeostasis (71, 72). For instance, (probiotic) *Lactobacillus* strains have been reported as potent producers of ROS that acts as a signal-transducing molecule *via* the PPARγ pathway in intestinal epithelial cells (73); PPARγ being a master regulator of energy metabolism and inducer of OXPHOS (74). The altered mucosal homeostasis is potentially regulated by sirtuin signalling, which is indicated by the activation of SIRT5 gene in the EF group, that is known to be exclusively located in mitochondria regulating energy metabolism and homeostasis (75, 76) as well as having potential immune regulatory (T cell activation and differentiation) functions (77, 78).

Weaning stress has been associated with altered gene expression of oxidative stress and immune pathways, regulated by MAPK signalling as a response to external stimuli (13, 14, 79). MAPK signalling are a class of mitogen-activated protein kinases that transduce signals from the cell membrane to the nucleus, leading to oxidative stress-induced differentiation, apoptosis, immune response. These response cascades were previously reported to be activated in pigs shortly after weaning (79–81). Our study corroborates these results and identifies p38 MAPK activation at day+3 along with the increased expression of immune-related genes (IL18, TGFB1, TGFB2) in EF compared to CON piglets. TGF-beta signalling is known to play an important regulatory role in intestinal barrier restoration by stimulating epithelial cell migration, extra-cellular matrix, integrin production, and can be important for post-weaning adaptation in pigs (82). Remarkably, EF piglets displayed an increased expression of TGFB1, TGFB2 as well as ECM (ACTG2, ACTN1, ACTN2, ITGA1, ITGA5, RHOD, RHOBTB1, VCL) genes, suggesting a relatively improved mucosa integrity. Effect on barrier integrity in EF piglets was also substantiated in jejunum with the strong downregulation of MMP-9 gene (Matrix metalloproteinase-9; involved in re-modelling of ECM and wound repair), that is known to cause an increase in intestinal epithelial tight junction permeability (83). Taken together, the EF piglets seem to be more adequately coping with the weaning-stress induced mucosal challenges by pronounced activation of barrier repair reactions, which is a combination of immune activation, epithelial migration and “wound-repair” like processes. The lack of activation in the CON piglets might reflect failure of the intestine to respond adequately to the stimulus of feed and/or microbiota triggered by weaning.

In agreement with previous studies (1, 84, 85), temporal changes induced by weaning were observed in jejunal mucosal morphology, characterised by altered villus length and crypt depth. Strikingly, EF piglets displayed a significantly higher jejunal villus length three days post-weaning (day+3) compared to the CON piglets, which suggests a lower “weaning dip” or a smoother weaning transition in terms of mucosal morphology. Furthermore, lowered villus length could be indicative of the less appropriate activation of barrier and tissue remodelling in the CON group, possibly reflected in the altered expression levels of proliferation, tissue-remodelling associated genes (RHOC, RHOD, RHOE, RAC3) in jejunum. Unlike earlier work that focussed on jejunal morphology, this study also assessed the temporal changes in colon mucosa and detected an increasing colonic crypt depth over time, though this process did not appear to be affected by early feeding. Of note, it is remarkable to observe the impact of early feeding on both microbiota and host mucosa, in spite of the relatively low and variable (fibrous) feed intake of (EF) piglets (**Supplementary Figure 9**) compared to the milk intake [approximately 28 grams/day/piglet from d21-30 in this study *vs*. 1 kg milk/day in (86)] that constitutes the main energy source of the piglet.

Performance parameters were also assessed in our study and a quite moderate smoothening of weaning transition was observed, reflected in the increased post-weaning feed intake (between day+1 and day+5), average daily gain (between day+1 and day+2) and consistently lowered coefficient of variation in body weight development (**Supplementary Figure 10**), which was also seen previously (42). However, we also observed a fluctuating average daily gain between the two treatment groups within 2 days post-weaning and no difference in relative body weight post-weaning, which makes the importance of early feeding in improving the postweaning performance, unclear. It is although important to note that these piglets were healthy and unchallenged, and therefore a more stressful situation (e.g., nutritional or environmental challenge models such as low-hygiene, heat stress, fasting) can possibly detect differences in coping adaptability between the groups.

In conclusion, the results from the present study confirmed the acceleration in maturation of the microbiota and clearly demonstrated that this “accelerated maturation” is also reflected in the host mucosal transcriptome in early-fed piglets. The accelerated changes in the EF piglets were closely associated with upregulation of cellular energy metabolism and immune tolerance potentially induced by the colonised commensals. Remarkably, the EF piglets seem to be more responsive to the post-weaning situation (day+3), which may contribute to the smoothening of the weaning stress by supporting better maintenance of adequate immune responses, gut barrier integrity and intestinal morphology. Of note, our study addresses fundamental questions about how the microbiome regulates host metabolism, which might aid in the understanding of dietary fibre-microbiota-host mucosa interactions in human infant studies, in addition to exploring early feeding as an attractive strategy to prepare suckling piglets for weaning transition.

## Data availability statement

The datasets presented in this study can be found in online repositories. The names of the repository/repositories and accession number(s) can be found below: https://www.ebi.ac.uk/ena, PRJEB61259 (ERP146358).

## Ethics statement

The animal study was reviewed and approved by The Animal Care and Use committee of Wageningen University Research (Wageningen, The Netherlands) (AVD104002016515).

## Author contributions

JB and MK conceived the study and acquired funding. RC performed the experiment, collected, and processed samples, analysed sequencing data, statistics, and prepared the figures. YG performed laboratory experiments related to histology and quantitative immunohistochemistry. RC and MK interpreted data, wrote the original draft of the manuscript. All authors discussed the results, commented on the manuscript, and approved the final manuscript. All authors contributed to the article.
