# Supplementary material for: Early feeding leads to molecular maturation of the gut mucosal immune system in suckling piglets
Source: Front Immunol. 2023 May 25;14:1208891. doi: 10.3389/fimmu.2023.1208891 (PMC10248722; doi:10.3389/fimmu.2023.1208891)
Supplement: Supplementary file 1 [file DataSheet_1.pdf]

Supplemental information belonging with:

Early feeding leads to molecular maturation of the gut mucosal immune system in suckling piglets

R. Choudhury, Y. Gu, J.E. Bolhuis, M. Kleerebezem

Supplementary Table 1: Ingredients and calculated nutrient composition of the pre-weaning fibrous feed[1]

	**Pre-weaning feed**
**Ingredients, %**	
Wheat	21.9
Barley	15
Maize	15
Soy protein concentrate	7
Soybeans (heat treated)	5
**Galacto-oligosaccharides[2]**	**5**
Potato protein	4
**Sugarbeet pulp**	**4**
**Oat hulls**	**4**
**Inulin[3]**	**4**
**Resistant starch[4]**	**4**
Soybean oil	3
Blood meal (spray dried)	2
Dicalcium phosphate	1.7
Sucrose	1.5
Calcium carbonate	1.0
Sodium chloride	0.5
Premix[5]	0.5
Potassium bicarbonate	0.3
L-lysine hydrochloride	0.3
DL-methionine	0.2
L-threonine	0.04
L-tryptophan	0.04
**Calculated nutrient composition, g/Kg**	
Dry matter	891
Starch	290
NSP[6]	261
Crude protein	195
Crude fat	61
Crude fibre	44
Crude ash	57
Calcium	9.1
Phosphorus	6.1
Sodium	2.2
Standardized ileal digestible lysine	11.9
Standardized ileal digestible methionine	4.8
Standardized ileal digestible threonine	7.1
Standardized ileal digestible tryptophan	2.4
Net energy, MJ/kg	11.8

[1] Feed was mixed and pelleted (12mm diameter pellets) by Research Diet Services (Wijk bij Duurstede, The Netherlands). According to CVB (2007), nutrients are presented in g/kg dry matter, except for dry matter (g/kg) and net energy (MJ/kg).
[2] Source: Vivinal® GOS powder (Friesland Campina, Amersfoort, The Netherlands) containing 69% galacto-oligosaccharides.
[3] Source: Prebiofeed 95 inulin powder (Cosucra group, Belgium) containing 85% inulin.
[4] Source: AmyloGel® Native Starches (Cargill, Wayzata, USA) derived from high amylose maize with 75% amylose content.
[5] Vitamin and mineral premix (per kg of feed): vitamin A: 10000 IU, vitamin D3: 2000 IU, vitamin E: 40 mg, vitamin K: 1.5 mg, vitamin B1: 1 mg, vitamin B2: 4 mg, vitamin B6: 1.5 mg, vitamin B12: 0.02 mg, niacin: 30 mg, D-pantothenic acid: 15 mg, choline chloride: 150 mg, folate: 0.4 mg, biotin: 0.05 mg, iron: 100 mg, copper: 20 mg, manganese: 30 mg, zinc: 70 mg, iodine: 0.7 mg, selenium: 0.25 mg, anti-oxidant: 125 mg.
[6] Non-starch polysaccharide: Calculated as the difference between dry matter and the sum of starch, sugars, crude protein, crude fat and crude ash.

Supplementary Table 2: Ingredients and calculated nutrient composition of the weaner feed[1], that closely resembles the composition of standard commercial weaner diets

	Post-weaning feed
**Ingredients, %**	
Barley	37.9
Wheat	20.0
Corn	5.0
Soybean meal	4.0
Soybeans full fat toasted	5.0
Corn	5.0
Whey powder	5.0
Sugarbeet pulp	1.5
Provisoy™[2]	10.0
Soybean oil	2.5
Premix[3]	0.5
Limestone	1.05
Monocalcium phosphate	0.9
Sodium chloride	0.4
Sodium bicarbonate	0.1
L-lysine	0.52
DL-methionine	0.22
L-threonine	0.24
L-tryptophan	0.08
L-valine	0.1
**Calculated nutrient composition, g/Kg**	
Dry matter	886
Crude ash	54
Crude protein	175
Crude fat	57
Crude fibre	33
Carbohydrates	573
Starch	398
Sugar	68
Neutral detergent fiber (NDF)	109
Acid Detergent Fiber (ADF)	47
Calcium	6.7
Phosphorus, total	5.6
Magnesium	1.5
Potassium	8.3
Sodium	2.2
Chloride	5.0
Standardized ileal digestible lysine	11.0
Standardized ileal digestible methionine + cysteine	6.5
Standardized ileal digestible threonine	6.9
Standardized ileal digestible tryptophan	2.4
Standardized ileal digestible valine	7.7
Net energy (MJ/Kg)	10.6

[1] Mixed and pelleted (3mm diameter) by Research Diet Services (Wijk bij Duurstede, The Netherlands).

[2] Provimi, Cargill, Rotterdam, The Netherlands.

[3] Vitamin and mineral premix (per kg of feed): vitamin A: 10000 IU, vitamin D3: 2000 IU, vitamin E: 40 mg, vitamin K: 1.5 mg, vitamin B1: 1 mg, vitamin B2: 4 mg, vitamin B6: 1.5 mg, vitamin B12: 0.02 mg, niacin: 30 mg, D-pantothenic acid: 15 mg, choline chloride: 150 mg, folate: 0.4 mg, biotin: 0.05 mg, iron: 100 mg, copper: 20 mg, manganese: 30 mg, zinc: 70 mg, iodine: 0.7 mg, selenium: 0.25 mg, anti-oxidant: 125 mg.

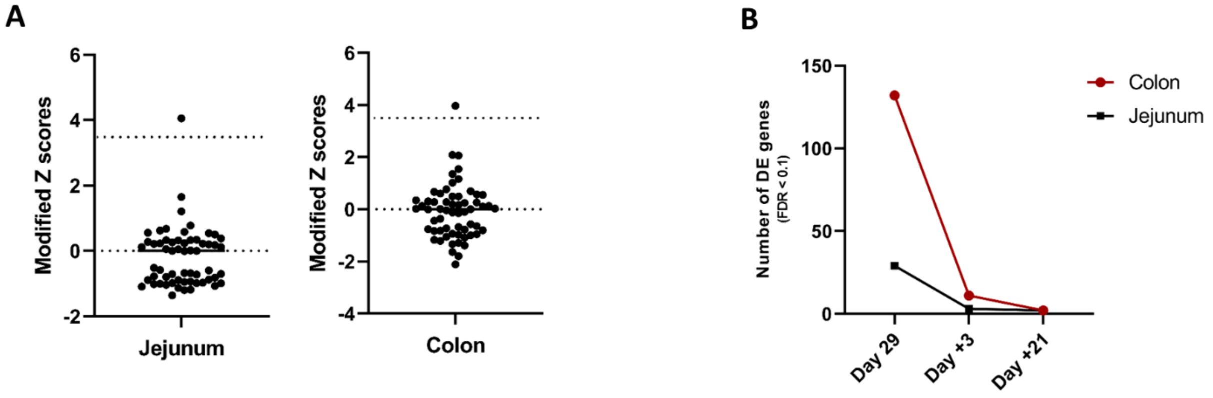

Supplementary figure 1: (A) Outliers were detected using modified Z scores of principal component loadings obtained from unsupervised principal component analysis in colon and jejunum samples. Samples exceeding absolute score of 3.5 are regarded as outliers. **(B)** Number of differentially expressed (DE) genes (EdgeR test: *FDR* < 0.1) at day29, day+3 and day+21 in colon and jejunal samples.

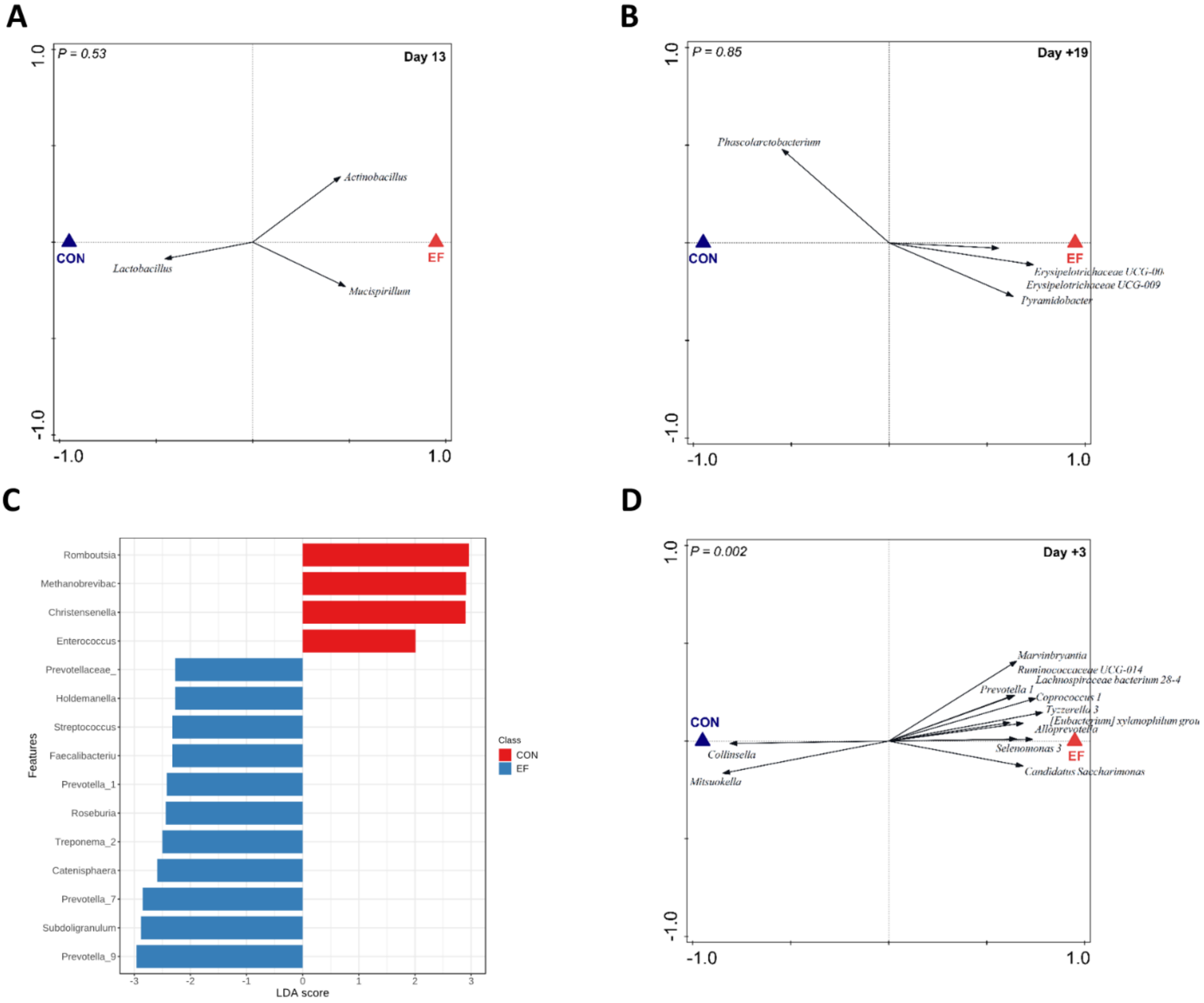

Supplementary figure 2: Microbiota composition (genus level) in early-fed (EF) and control (CON) group. **(A)** Redundancy analysis of EF and CON treatment groups at day 13 (swabs; explained variation = 0.00%; *P* = 0.53). **(B)** Redundancy analysis of treatment groups at day+19 (swabs; explained variation = 0.00%; *P* = 0.85). **(C)** LEfSe analysis (colon, d29) identified the differentially abundant microbial groups between EF and CON. **(D)** Redundancy analysis (colon, d+3) of treatment groups (explained variation = 9.8% ; *P* = 0.002).

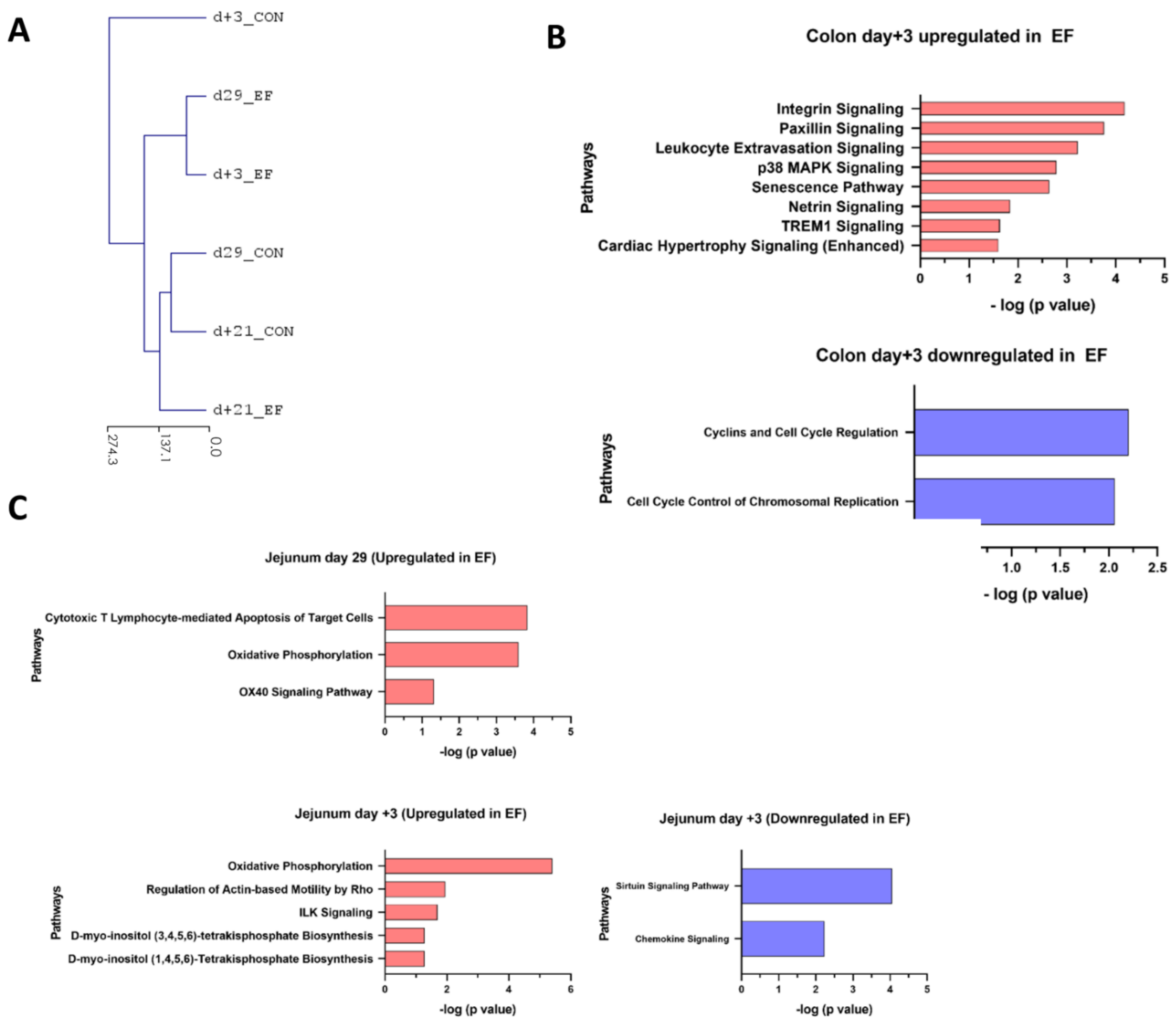

Supplementary figure 3: (A) Unsupervised hierarchical clustering (euclidean distance) of jejunal transcriptome using normalised (logCPM) expression values (averaged per group per time-point) and are scaled by the mean value of day+21 gene expression (irrespective of treatment). Pathway analysis in **(B)** Colon and **(C)** Jejunum. Canonical pathways up and downregulated in early-fed (EF) group compared to the control (CON) group are shown in red and purple bars respectively. Identified pathways have a logP value ≥ 1.3 (enrichment score from Fisher's exact test, Ingenuity pathway analysis) and an absolute Z score ≥ 2 (assessing the match of observed and predicted up/down regulation patterns).

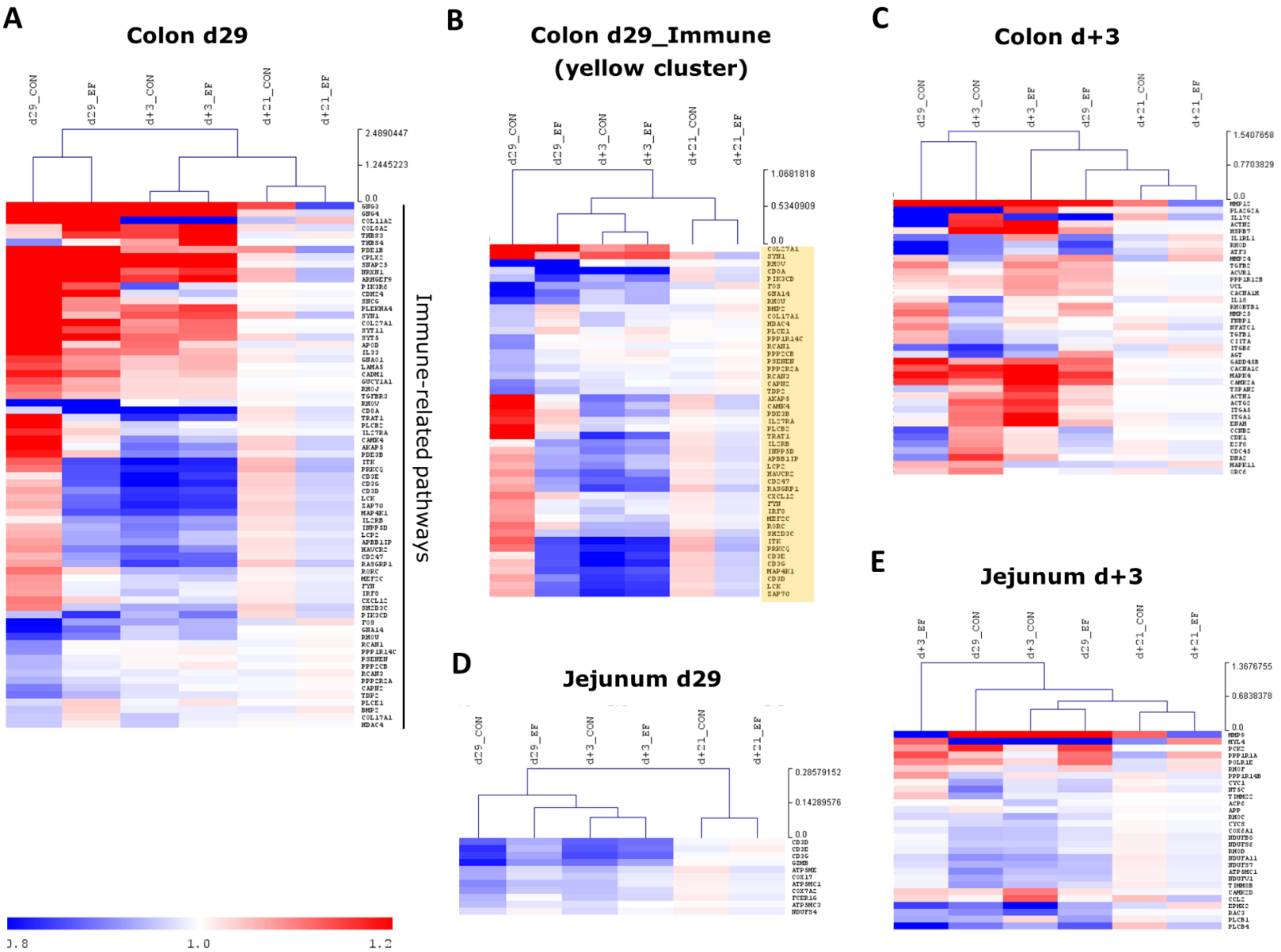

Supplementary figure 4: Hierarchical clustering of expression profiles over time for (**A**) immune pathway genes of colon at day29. (**B**) the third cluster of immune genes (yellow) observed in **Figure 5C** (**C**) colon day+3 (upregulated) (**D**) jejunum day29 (**E**) jejunum day+3. The normalised expression values (averaged per group per time-point) are scaled by the mean value of total day+21 expression (irrespective of treatment). EF = early-fed, CON = control.

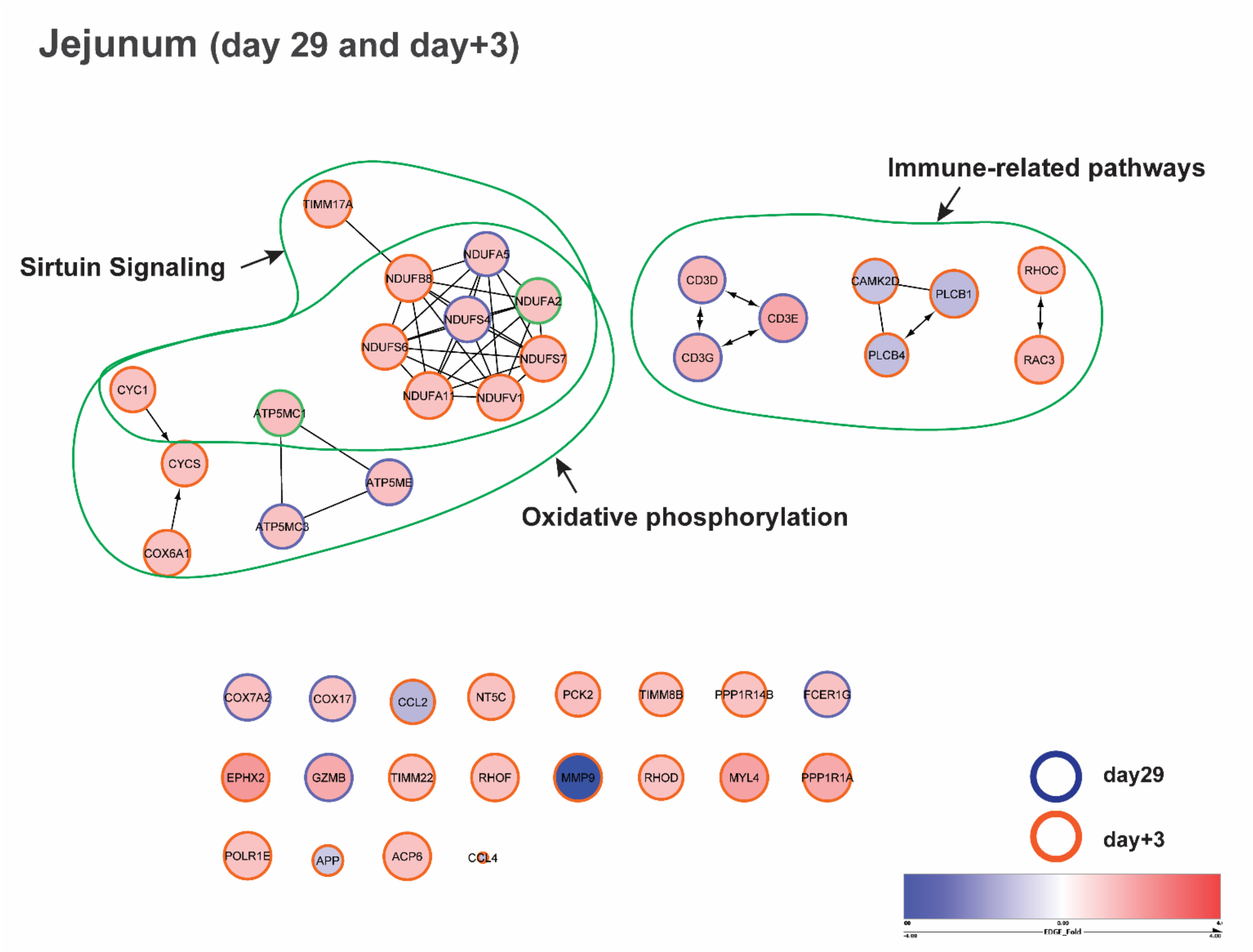

Supplementary figure 5: Functional network of pathway identified genes (jejunum) at day29 and day+3. Functional protein-protein interaction of up- and down-regulated genes in early-fed (EF) compared to control (CON) group. The circles or nodes represent genes and edges represent interactions between genes, as determined by Reactome. Arrows represent directed interactions, bar-headed arrows indicate inhibition reactions and dotted lines indicate predicted relationships. Pathway identified genes from time-points day29 (blue bordered), day+3 (orange bordered) and both (green bordered) are shown in this figure. The upregulated (red nodes) and down-regulated (blue nodes) genes are represented by their fold change (EdgeR test) in a blue-red gradient scale, where the size of the node is proportional to their significance (P value; EdgeR test). The dotted green lines depict different pathways associated with those genes. Two genes ATP5MC1 and NDUFA2 (part of oxidative phosphorylation pathway) were observed at both day29 and day+3 (fold change ~ 1.3; $P < 0.05$).

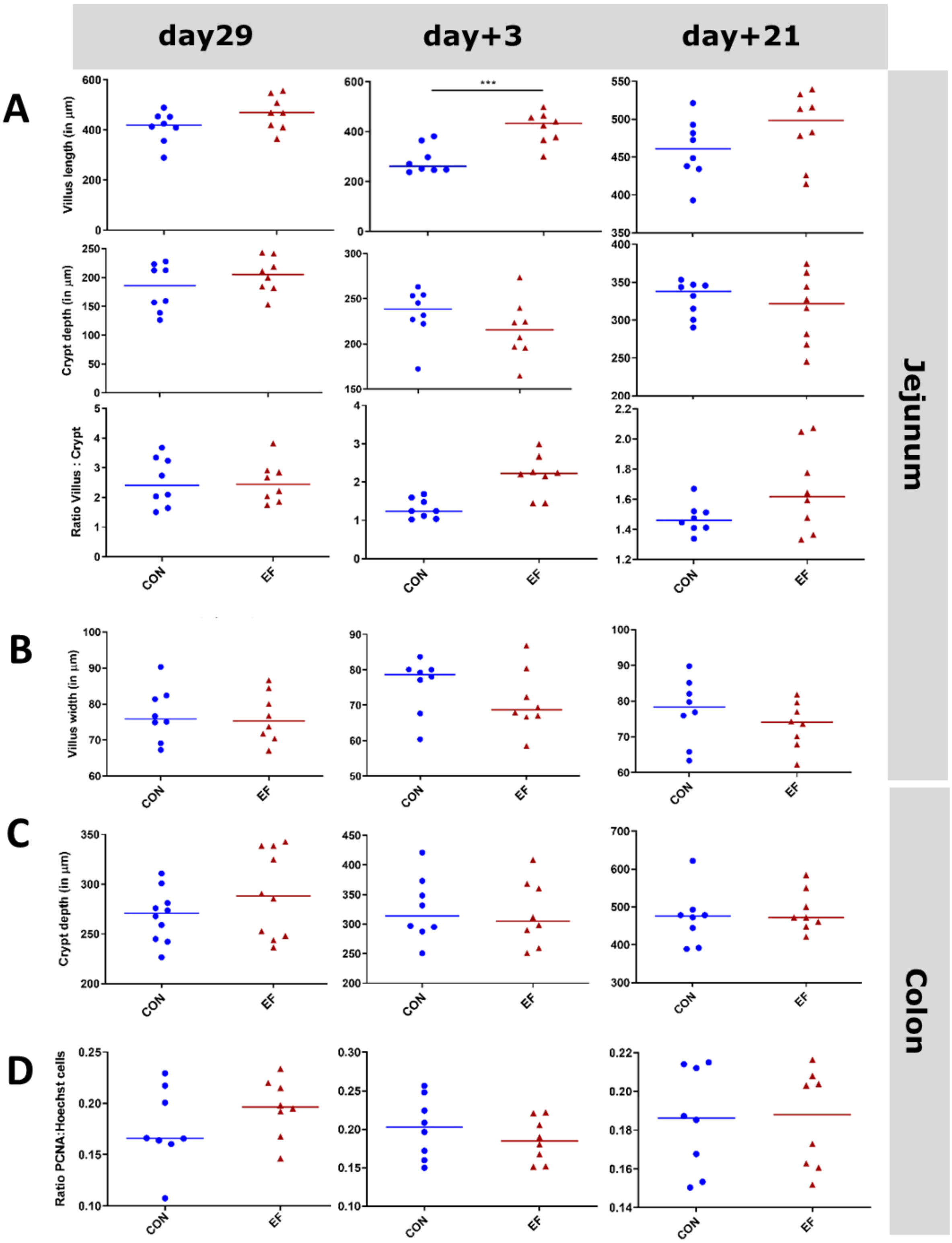

Supplementary figure 6: Jejunal and colonic morphometric measurements determined at three sampling time-points indicated at the top (day29, day+3, and day+21) for individual piglets in the early-fed (EF; red) and control (CON; blue) groups. **Panel A:** Jejunal villus length (upper panels), crypt depth (middle panels) and villus:crypt ratio (lower panels). **Panel B:** jejunal villus width, **Panel C:** colonic crypt depth, and **Panel D**: colonic ratio of PCNA over Hoechst stained nuclei, indicative of relative epithelial proliferation activity. Differences between groups were assessed by t-test or Mann-Whitney U test (non-parametric).

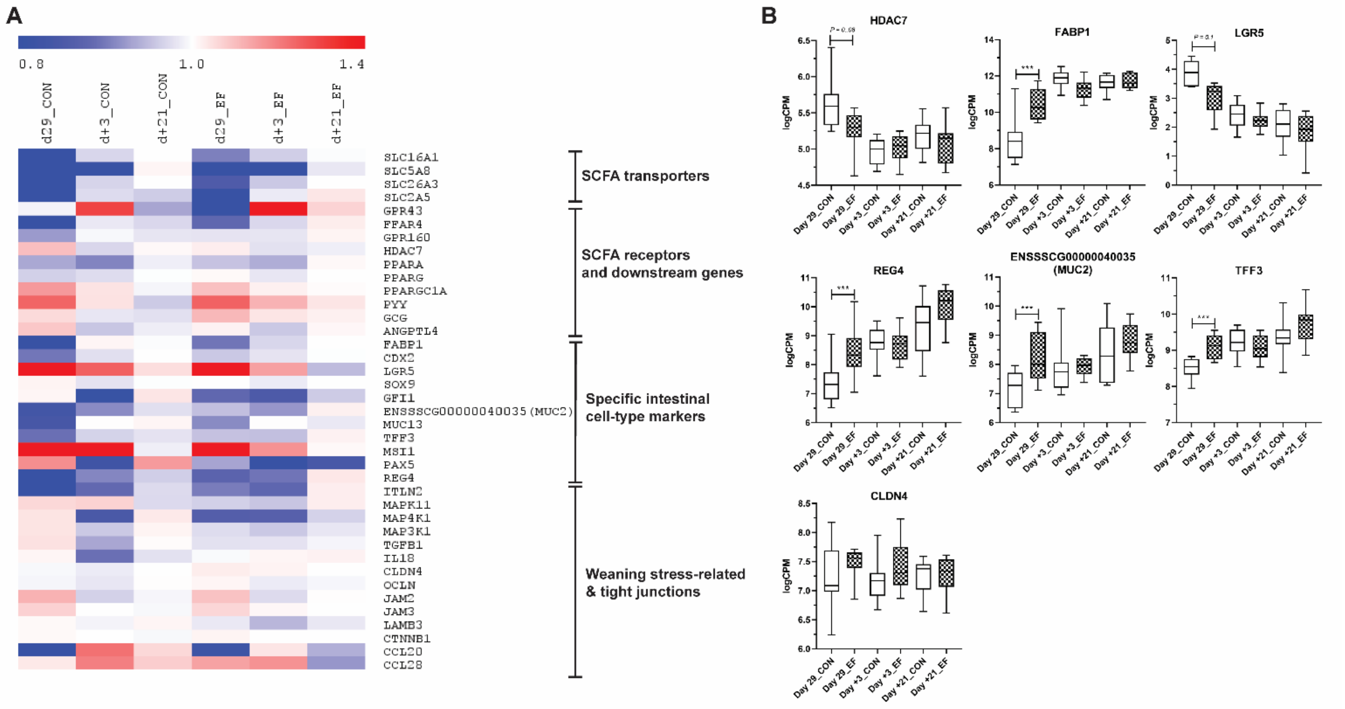

Supplementary figure 7: (**A**) Heatmap displaying colonic gene expression of SCFA transporters, SCFA sensing receptors, specific intestinal cell-type markers and tight junction proteins (literature identified) in CON and EF piglets. The expression values (averaged per group per time-point) are scaled by the mean value of total day+21 expression (irrespective of treatment). (**B**) A few genes shown in box plots along with their P value (differential expression analysis, EdgeR test) (***: P < 0.001).

The maturation of the EF piglets was apparent from the modulation of certain markers (differential expression of marker genes) for specific cell-types such as FABP1 (fatty acid-binding protein 1; associated with differentiated enterocytes), CDX2 (caudal type homeobox 2; associated with enterocyte differentiation and proliferation), LGR5 (leucine rich repeat containing G protein-coupled receptor 5; stem cell marker), TFF3 (trefoil factor 3; expressed within goblet cells) as well as REG4 (regenerating family member 4; deep crypt secretory cells functioning as the colon equivalent of Paneth cells) as well as MUC2 gene (common goblet cell marker), indicating the maturation of EF piglets. The differential expression between the treatment groups, however, did not hold true for other known Paneth and goblet cell markers such as, SOX 9 (SRY-Box transcription factor 9; involved in Paneth and goblet cell differentiation), GFI1 (growth factor independent 1 transcription repressor), MSI1 (musashi RNA binding protein 1; suppresses Paneth cell differentiation).

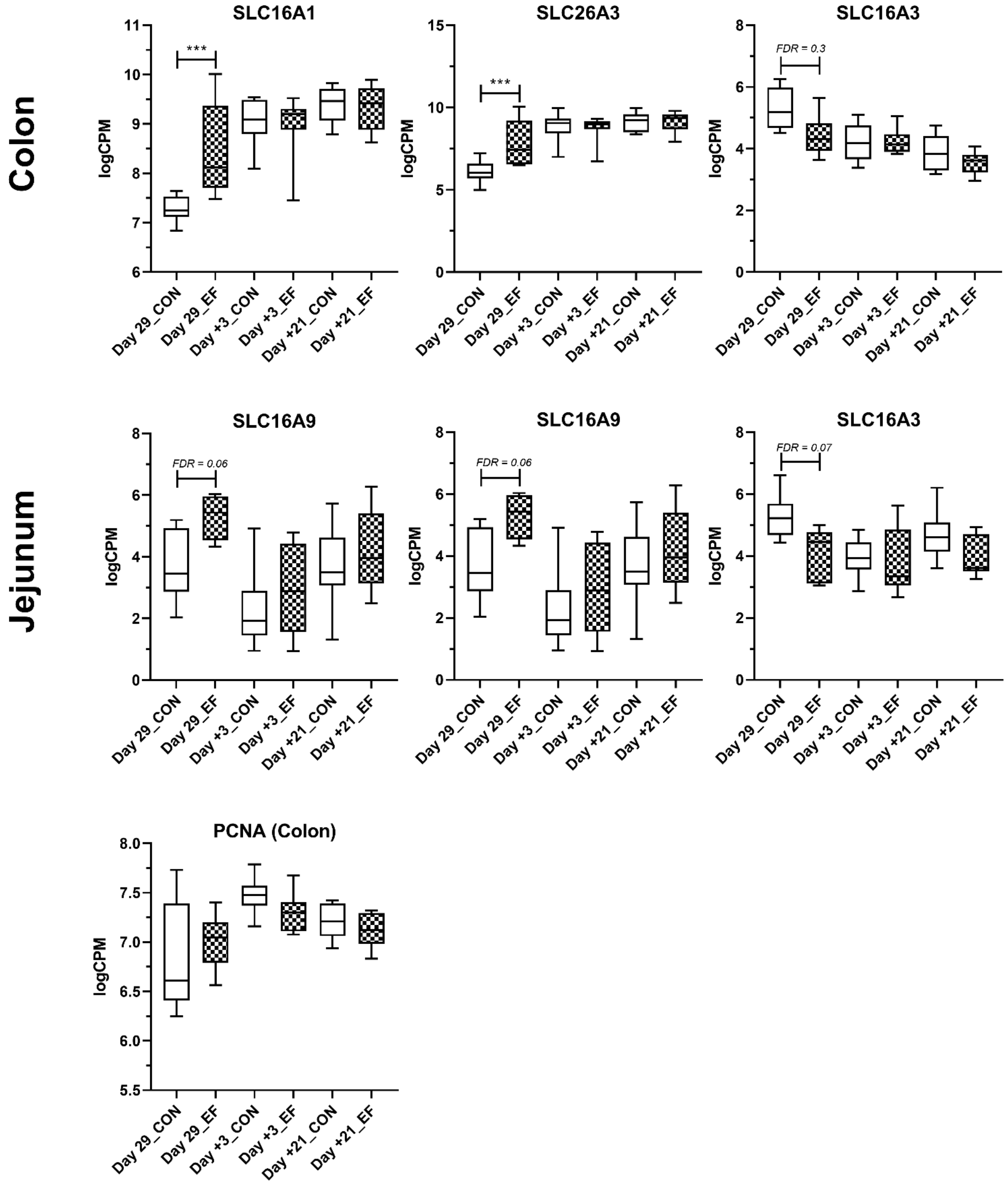

Supplementary figure 8: Gene expression levels of SCFA transporters (belonging to solute carrier family) that were found significantly altered in early-fed (EF) as compared to the control (CON) piglets (EdgeR test, P < 0.05). In addition, gene expression levels of (colon) proliferating marker PCNA is shown.

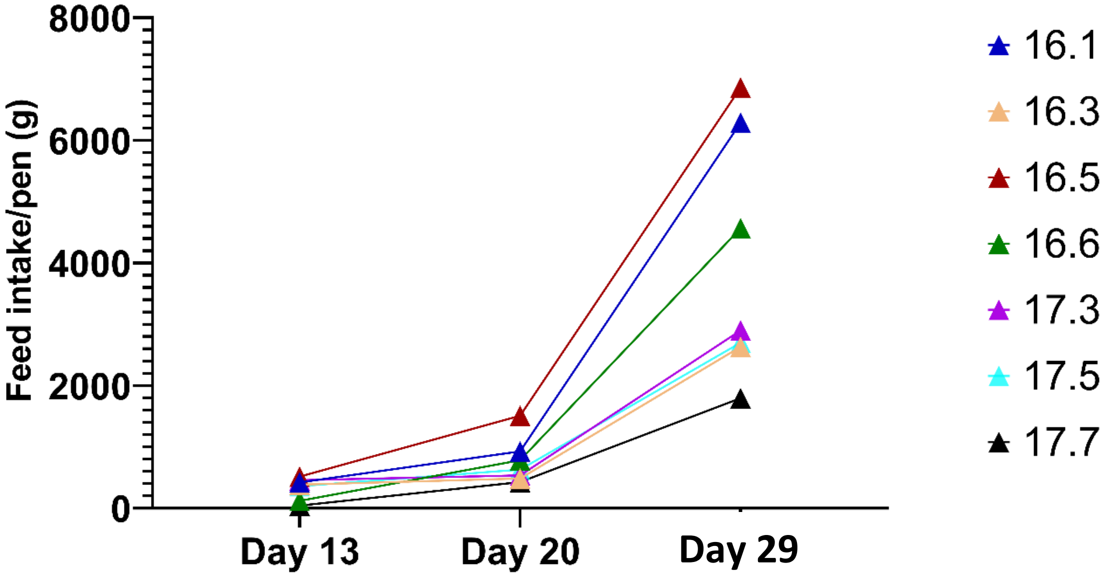

Supplementary figure 9: Pen-level pre-weaning feed intake (g) in early-fed (EF) piglets, measured at three timepoints. The seven EF pens are shown in colours.

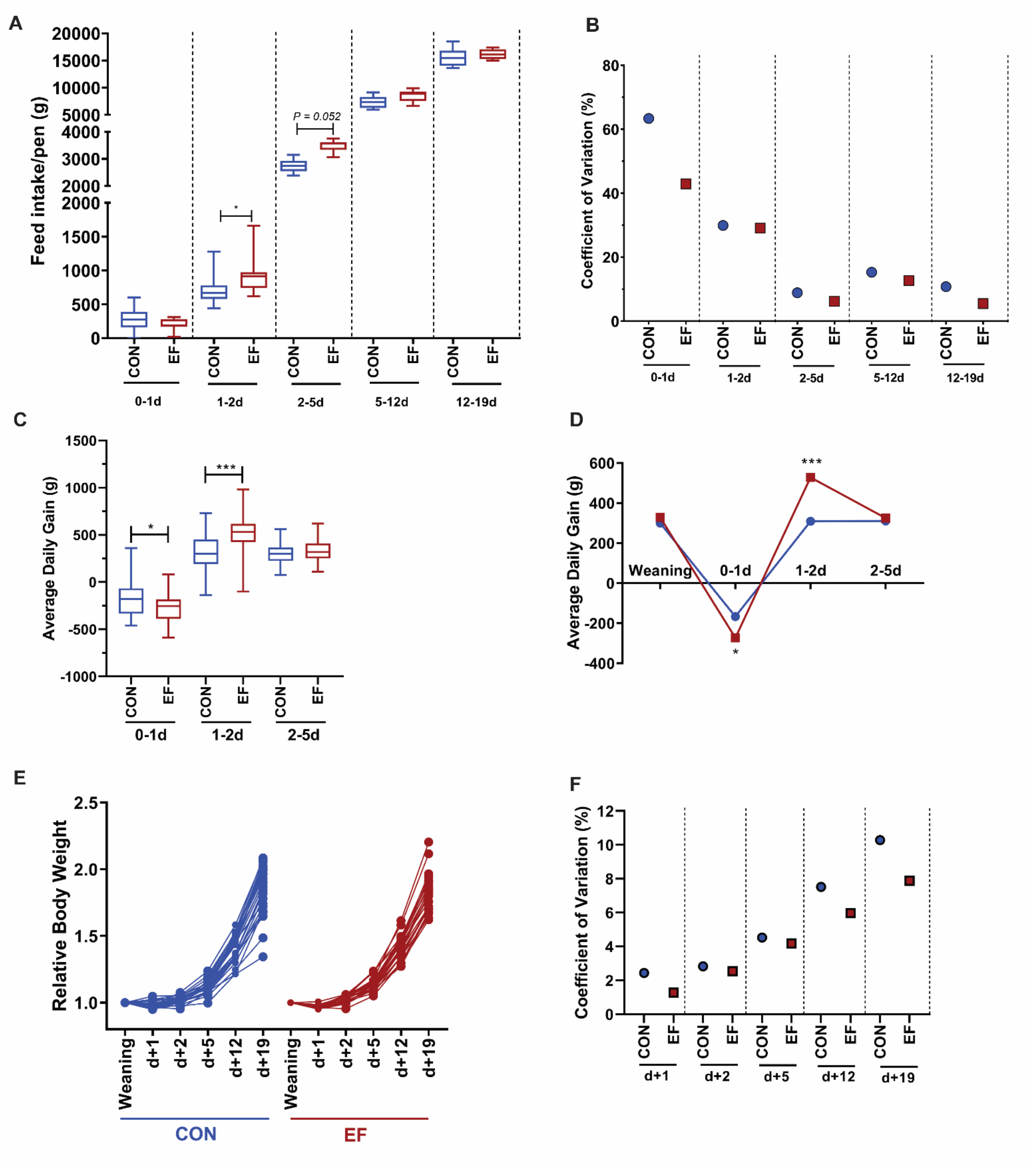

Supplementary figure 10: (**A**) Pen-level post-weaning feed intake in control (CON; blue) and early-fed groups (EF; red) measured between 0-1 day, 1-2 day, 2-5 days, 5-12 days and 12-19 days post-weaning. All pens were provided with weaner diet after weaning (n = 12 pens per treatment group till day+2, after which n = 8 per treatment till day+19 due to sacrifice of piglets). (**B**) Coefficient of variation (%) which captures the dispersion of the feed intake data at each time-point per treatment. (**C**) Average daily gain (ADG, in grams; n = 24 per treatment group) in the early-post-weaning period. (**D**) Mean of the ADG shown during the first 5 days post-weaning, comparing with the pre-weaning (three days before weaning) ADG per treatment. (**E**) Relative body weight development (relative to weaning weight per individual piglet) between the groups (n = 48 per treatment) till 19 days post-weaning. (**F**) The coefficient of variation (CV%) capturing the variation in body weight in CON and EF groups. Statistical comparisons between the groups were assessed by student t test or Mann-Whitney U test in GraphPad Software 8.1.1.
